# Evidence on the inhibitory effect of *Brassica* plants against *Acinetobacter baumannii* lipases: phytochemical analysis, in vitro, and molecular docking studies

**DOI:** 10.1186/s12906-024-04460-y

**Published:** 2024-04-19

**Authors:** Manal M. Sabry, Ali M. El-Halawany, Walaa G. Fahmy, Basma M. Eltanany, Laura Pont, Fernando Benavente, Ahmed S. Attia, Farag F. Sherbiny, Rana M. Ibrahim

**Affiliations:** 1 Department of Pharmacognosy, Faculty of Pharmacy, Cairo University, Cairo, 11562 Egypt; 2 Department of Microbiology & Immunology, Faculty of Pharmacy, Cairo University, Cairo, 11562 Egypt; 3 Department of Pharmaceutical Analytical Chemistry, Faculty of Pharmacy, Cairo University, Cairo, 11562 Egypt; 4 Department of Chemical Engineering and Analytical Chemistry, Institute for Research on Nutrition and Food Safety (INSA·UB), University of Barcelona, Barcelona, 08028 Spain; 5 Serra Húnter Program, Generalitat de Catalunya, Barcelona, 08007 Spain; 6 School of Pharmacy, Newgiza University, Giza, 12577 Egypt; 7 Pharmaceutical Organic Chemistry Department, Faculty of Pharmacy, Al-Azhar University, Nasr city, Cairo, 11884 Egypt

**Keywords:** *Acinetobacter baumannii*, *Brassica*, Docking study, Lipase inhibition, Mass spectrometry

## Abstract

**Background:**

Infections caused by *Acinetobacter baumannii* are becoming a rising public health problem due to its high degree of acquired and intrinsic resistance mechanisms. Bacterial lipases penetrate and damage host tissues, resulting in multiple infections. Because there are very few effective inhibitors of bacterial lipases, new alternatives for treating *A. baumannii* infections are urgently needed. In recent years, *Brassica* vegetables have received a lot of attention since their phytochemical compounds have been directly linked to diverse antimicrobial actions by inhibiting the growth of various Gram-positive and Gram-negative bacteria, yeast, and fungi. Despite their longstanding antibacterial history, there is currently a lack of scientific evidence to support their role in the management of infections caused by the nosocomial bacterium, *A. baumannii*. This study aimed to address this gap in knowledge by examining the antibacterial and lipase inhibitory effects of six commonly consumed *Brassica* greens, Chinese cabbage (CC), curly and Tuscan kale (CK and TK), red and green Pak choi (RP and GP), and Brussels sprouts (BR), against *A. baumannii* in relation to their chemical profiles.

**Methods:**

The secondary metabolites of the six extracts were identified using LC-QTOF-MS/MS analysis, and they were subsequently correlated with the lipase inhibitory activity using multivariate data analysis and molecular docking.

**Results:**

In total, 99 metabolites from various chemical classes were identified in the extracts. Hierarchical cluster analysis (HCA) and principal component analysis (PCA) revealed the chemical similarities and variabilities among the specimens, with glucosinolates and phenolic compounds being the major metabolites. RP and GP showed the highest antibacterial activity against *A. baumannii*, followed by CK. Additionally, four species showed a significant effect on the bacterial growth curves and demonstrated relevant inhibition of *A. baumannii* lipolytic activity. CK showed the greatest inhibition (26%), followed by RP (21%), GP (21%), and TK (15%). Orthogonal partial least squares-discriminant analysis (OPLS-DA) pinpointed 9 metabolites positively correlated with the observed bioactivities. Further, the biomarkers displayed good binding affinities towards lipase active sites ranging from −70.61 to −30.91 kcal/mol, compared to orlistat.

**Conclusion:**

This study emphasizes the significance of *Brassica* vegetables as a novel natural source of potential inhibitors of lipase from *A. baumannii*.

**Supplementary Information:**

The online version contains supplementary material available at 10.1186/s12906-024-04460-y.

## Background

Brassicaceae is made up of 350 genera, including *Camelina*, *Crambe*, *Sinapis*, *Thlaspi*, and *Brassica*, which have over 3,500 species. *Brassica* is the most important genus, encompassing crops and species of great worldwide economic value such as cabbage, cauliflower, and broccoli. *Brassica* crops have sparked scientific interest due to their potential applications in treating various cardiovascular ailments and gastrointestinal cancers [1]. Additionally, the antioxidant capacity and antibacterial activity of several *Brassica* vegetables, such as kale, Brussels sprouts, cabbage, broccoli, and radish have been previously studied [2–5].

Sulfur-containing compounds, glucosinolates, are the main identified compounds in *Brassica* vegetables and they are responsible for their characteristic aroma [6] and various biological activities [7]. Several studies have also reported the existence of polyphenolics as major constituents in *Brassica* leaves [8, 9]. The most commonly reported flavonoids are quercetin, kaempferol, and isorhamnetin, which are glycosylated and/or acylated with one or more hydroxycinnamic acids, including coumaric, caffeic, sinapic, and ferulic acids [8, 10]. Several reports correlated the high polyphenolic content of *Brassica* leaves to their antioxidant, anticancer, antihyperglycemic, and hepatoprotective potential [11–14]. The bioactive potential of Brassicaceae glucosinolates and isothiocyanates is obvious, making them great candidates for biocontrol of pathogens that cause severe illnesses in humans [15]. They may have inhibitory activity against a wide range of microorganisms, including fungi and pathogenic bacteria like *Escherichia*, *Salmonella*, *Bacillus*, *Staphylococcus*, *Klebsiella*, and *Listeria*, or in synergism with conventional antibiotics to enhance their effectiveness [15, 16]. Other studies found that isothiocyanates derived from *B. campestris* ssp. *pekinensis* and *B. rapa* var. *rapa*, such as benzyl, 3-butenyl, and 2-phenyl ethyl isothiocyanate had stronger antibacterial effects against Gram-positive bacteria than Gram-negative bacteria when tested using the agar disc diffusion assay [17]. Furthermore, the antibacterial and antioxidant activities of several *Brassica* phenolics were previously discussed [3, 9]. Different flavonoids in cabbage, such as genistein, kaempferol, naringenin, and catechin revealed potent antibacterial activity against the *Staphylococcus aureus* and *Escherichia coli* [9].

Metabolomics aims at the identification and quantification of small molecules in biological samples, such as plant materials [18]. Recently, LC-MS/MS has gained popularity as the preferred platform for metabolomic studies due to its high throughput, mass accuracy, resolution, detailed characterization, and broad coverage of metabolites [19]. LC-MS/MS metabolomics analyses are combined with multivariate analysis methods, in particular, principal component analysis (PCA) and orthogonal partial least squares-discriminant analysis (OPLS-DA) to uncover chemical variations among groups of samples, providing an effective route for the subsequent identification of bioactive components [20, 21]. Nevertheless, previous research on *Brassica* species has focused on targeting specific classes of constituents, such as polyphenolic compounds [8, 10, 22] or glucosinolates [23–25]. In our study, we apply this LC-MS/MS-based metabolomics approach to obtain comprehensive profiles of both secondary and primary metabolites in these economically valuable vegetables. Moreover, to add a biological dimension to our findings, the antibacterial activity of the selected *Brassica* species was evaluated against *Acinetobacter baumannii*. This *Gram*-negative pathogen is notorious for its impact on vulnerable intensive care unit patients, leading to severe infections such as ventilator-associated pneumonia and bacteremia. These infections pose a significant threat in developing countries [26]. *A. baumannii* is an emerging pathogen that currently holds the top position on the World Health Organization (WHO) list of pathogens in urgent need of new effective antibacterial agents [27]. Unfortunately, the treatment of *A. baumannii* is often complicated due to its inherent resistance to many commonly used antibiotics, as well as its ability to become resistant to numerous others. This resulted in significant outbreaks of multi-drug resistance (MDR) strains, including resistance to last-resort antibiotics such as colistin and tigecycline [28]. As far as the authors know, the antibacterial and anti-virulence activity of *Brassica* leaves against *A. baumannii* remains unexplored.

Molecular docking serves as a valuable tool in drug discovery programs, especially for natural products, by predicting interactions of small molecules with drug targets [29]. This process guides synthesis decisions, aids in understanding some traditional medicinal plant applications, and identifies new ones. Moreover, it reduces the effort required to isolate active compounds for structure elucidation studies and minimizes the need for bioactivity assessments, which are significant challenges in developing countries [29, 30, 31]. In summary, this study emphasizes the importance of *Brassica* greens as a natural source of antibacterial and anti-virulence compounds with potential implications for the food and nutraceutical industries.

This study presents the first comparative untargeted metabolomics approach, utilizing liquid chromatography-quadrupole time-of-flight tandem mass spectrometry (LC-QTOF/MS/MS) combined with multiple chemometrics tools, for correlating the chemical profiles of *Brassica* greens, including Chinese cabbage (CC), Curly (CK) and Tuscan kale (TK), green (GP), red Pak choi (RP), and Brussels sprouts (BR) to their antibacterial and lipolytic activities against *A. baumannii*. The findings were further validated using molecular docking to predict possible binding conformations of the different biomarkers with *A. baumannii* lipase active sites and to unravel the mode of interactions underlying the predicted lipolytic inhibitory effects of *Brassica* phytochemicals for the first time.

## Materials and methods

### Plant material

Six *Brassica* species were supplied by Makar Farm (Giza, Egypt) in March 2021 and kindly identified by Prof. Dr. Abdel-Halim Mohammed (Professor of Agriculture, Flora department, Agricultural Museum, Dokki, Giza, Egypt). Samples were kept in the Museum of Pharmacognosy Department, Faculty of Pharmacy, Cairo University. Voucher specimen numbers were 06032021I for *Brassica rapa* ssp. *chinensis* (Chinese cabbage (CC)), 07032021X for *B. olearacea* var. *Sabellica* (curly kale (CK)), 06032021II for *B. olearaceae* var. *palmifolia* (Tuscan kale (TK)), 07032021 IV for *B. rapa* ssp. *pekinensis* (green Pak choi (GP) and red Pak choi (RP)), and 07032021 VIII for *B. olearaceae* var. *gemmifera* (Brussels sprouts (BR)).

### Plant material extraction

Forty grams of the air-dried leaves of each *Brassica* species were extracted three times with one liter of methanol assisting by sonication. Each extract was then dried under vacuum in a rotary evaporator at 50 ºC and the residue was kept in a tight container until analysis. Each sample species was prepared as three biological replicates under the same conditions. Samples for LC-QTOF-MS/MS were prepared by dissolving 10 mg of each extract in 1 mL of methanol followed by centrifugation at 13,250 g for 10 min at 25 ºC and filtration through 0.22 nm syringe filter.

### LC-QTOF-MS/MS analysis

All LC-QTOF-MS/MS experiments were performed in a 1260 Infinity liquid chromatograph coupled to a 6546 LC/QTOF mass spectrometer with an orthogonal electrospray ionization (ESI) interface (Agilent Technologies, Waldron, Germany). Acetonitrile (ACN), water, methanol (LC-MS grade), and formic acid (≥ 95.0%) were supplied by Merck (Darmstadt, Germany). The chromatographic separation and MS detection conditions were reported in our previous work [20, 21].

Identification of the detected metabolites was achieved based on the exact mass, MS/MS fragmentation pattern, and molecular formula along with the current literature. The raw LC-QTOF-MS/MS data was converted with ProteoWizard 3.0 (www.proteowizard.org) into mzXML format and processed using MZmine 2.53 software (https://github.com/mzmine/mzmine2/releases/tag/v2.53) [32]. The obtained peak lists (3697 detected compounds) were then aligned and subjected to formula prediction and identification using LipidMaps (https://www.lipidmaps.org/), KEGG (https://www.kegg.jp/kegg/compound/), and MS/MS fragmentation patterns (99 identified metabolites). The data of the identified metabolites was then converted into a CSV file, containing the ID number (ID), retention time (t_r_), *m/z*, peak intensity, molecular formula, and metabolite identity in the different samples. The data set with the intensity information (18 columns: 6 *Brassica* species x 3 replicates) and 3697 rows (detected compounds) was then mean-centered, and exported to SIMCA Version 14 (Umetrics, Umeå, Sweden), for further analysis using hierarchical cluster analysis (HCA), principal component analysis (PCA), and orthogonal partial least squares-discriminant analysis (OPLS-DA).

### Microbiological analysis

#### Determination of the minimum inhibitory concentration (MIC)

The minimum inhibitory concentration (MIC) of extracts against the multidrug-resistant (MDR) *A. baumannii* strain AB5075 was determined using the microdilution method, following established protocols [33, 34]. Extracts were dissolved in DMSO and serially diluted in saline to calibrate in the concentration range between 1024 µg/mL and 0.5 µg/mL. *A. baumannii* cultures were grown to the mid-logarithmic phase, adjusted to a standardized density (0.5 McFarland’s standard), and exposed to the extracts in 96-well microtiter plates. Bacterial growth was assessed after 24 h incubation, and the MIC was defined as the lowest extract concentration that prevented visible growth. Orlistat served as a control.

#### Determination of the effective killing concentration

To determine the effective killing concentration of the orlistat and the studied extracts, especially those that showed turbidity with MHB, the assay described by the WHO [27] was adopted. The principle of this assay is that tetrazolium salts can form highly colored products upon reduction by viable biological systems. In the case of 2, 3, and 5-triphenyl tetrazolium (TTC), it is reduced to red tri-phenyl-formazan. Accordingly, the effective killing concentration can be considered as the lowest concentration that prevents the production of red tri-phenyl-formazan [35]. Briefly, the bacterial inoculum was prepared as described above for the MIC determination. Then, 15 µL aliquots corresponding to ∼ 5 × 10^5^ CFU were added to each well in a 96-well U-shaped microtiter plate. Then 150 µL of MHB with the tested extract was added to give a final extract concentration ranging from 420 µg/mL to 640 µg/mL, followed by incubation for 18 h at 37 °C. Positive and negative controls were included as described before for MIC experiments. Then, a 0.5% (m/v) solution of TTC (Sigma-Aldrich) was added to all the wells at a final concentration of 10% (v/v) and then incubated for 2 h at 37 °C. Finally, the wells that showed red color formation indicated the presence of viable cells.

#### Growth curves

The in vitro growth extent and rate of *A. baumannii* in the presence of the studied extracts were evaluated by establishing growth curves [36]. Briefly, the OD of an overnight bacterial culture was adjusted at OD600 ∼ 0.5 and then diluted to 1:100 (v/v) in fresh LB. The culture was then separately mixed with the extracts to a final concentration of 512 µg/mL for both BR and CC and 256 µg/mL for the other 4 species. DMSO was also included as a control in equivalent amounts as in the extract. Cultures were incubated at 37 °C while shaking at 180 rpm. Aliquots from each culture were withdrawn and their OD600 were measured every hour for 10 h and finally after 24 h. Measurements were used for the construction of growth curves by plotting OD600 values versus time.

#### Lipolytic activity assay

Lipolytic activity assay was conducted according to the method described by Martínez and co-workers [37] with some modifications. *A. baumannii* was grown to the mid-logarithmic phase (OD600 ∼ 0.6). The adjusted cultures were supplemented with the studied extracts and then mixed with an equal volume of the substrate solution. The latter consisted of 2 mM p-nitrophenyl palmitate (pNPP) (Sigma-Aldrich) in 50 mM Tris-HCl (pH 7.2, Sigma-Aldrich) containing 2% ACN (Sigma-Aldrich). The final concentration of the extracts in the assay mixture was 512 µg/mL for both BR and CC and 256 µg/mL for the other 4 species. An aliquot of the reaction mixture was transferred to a flat-bottomed 96-well plate and the p-nitrophenol (pNP) released after pNPP hydrolysis was determined by measuring the absorbance at 410 nm both at time zero and after 3 h of incubation at 37 °C. Samples incubated with the equivalent amounts of DMSO were used as controls.

### Molecular docking

The identified biomarkers were subjected to molecular docking to recognize their binding modes and free energies of binding towards *A. baumannii* lipase receptors. The crystallographic structure of *A. baumannii* lipase was retrieved from Protein Data Bank (PDB ID: 5L2F). Docking analysis was performed using the AutoDock program [38]. AutoDock is a freely available suite of automated docking tools, which allows flexible ligand docking (http://autodock.scripps.edu). The 2D chemical structures of the investigated compounds and co-crystalized ligands were sketched using ChemBioDraw Ultra 14.0. To assess the efficacy of the docking method, we performed molecular redocking of co-crystalized ligands, which were validated by getting low root mean square deviation (RMSD) values between the docked and X-ray structures. Then, the co-crystalized ligand and the tested compounds were docked using the default protocol parameters. The docking results from the AutoDock program were further analyzed and visualized using Pymol software to investigate the putative interaction mechanism.

## Results and discussion

### Metabolite identification by LC-QTOF-MS/MS

The six *Brassica* leaf extracts were analyzed using LC-QTOF-MS/MS in negative ESI mode to obtain comprehensive, characteristic, and detailed metabolic fingerprints of the different species. Supplementary Fig. S1 shows representative full-scan MS base peak chromatograms for the six species. A total of 99 compounds were tentatively identified based on the exact mass, MS/MS fragmentation pattern, and molecular formula along with the current literature. The identified metabolites belonged to five chemical classes (glucosinolates, isothiocyanates, phenolic acids, flavonoids, organic, and fatty acids). A list of the identified metabolites, including the ID, Rt, *m/z* of the detected molecular ion and errors (< 6.2 ppm), molecular formula, and MS/MS fragments are given in Table S1. The fragmentation patterns of selected identified metabolites are displayed in the supplementary material (Figs. S2–S11).

#### Glucosinolates (GSL)

GSL are amino acid-derived compounds with an O-sulfated thiohydroximate attached to a β-D-glucopyranoside moiety through a thio-link [39, 40]. Based on the nature of the parent amino acid, GSL can be classified into three groups: aliphatic, indole, and aromatic GSL [40]. They are mainly present in plants of order Brassicales, in particular family Brassicaceae [41]. Fifteen GSL were identified across the six studied *Brassica* species, including eleven aliphatic GSL (IDs 1, 2, 3, 4, 6, 7, 8, 9, 10, 11, and 14), three indole GSLs (IDs 12, 13, and 15), and one aromatic GSL (ID 5) (Table S1 and Fig. S1).

The identified GSLs were eluted in the first seven minutes of the chromatographic run. Simple aliphatic GSL as sinigrin (ID 1) (Fig. S2), progoitrin (ID 3), and gluconapin (ID 9) exhibited the deprotonated molecular ions [M-H]^−^ at *m/z* 358.0269, 388.0381, and 372.0433, respectively. They produced characteristic ion peaks at *m/z* 259 corresponding to thioglucosyl [C_6_H_11_O_9_S]^–^ moiety, and *m/z* 96 corresponding to the hydrogen sulfate [HSO_4_]^–^ moiety [20]. Furthermore, glucobrassicanapin (ID 11) exhibited a deprotonated molecular ion at *m/z* 386.0588, fragment ions at *m/z* 306 [M-H-SO_3_]^−^, 274 [C_6_H_10_O_8_S_2_]^−^, 259 [C_6_H_11_O_9_S]^–^, 95 [SO_4_]^–^, and 74 [OH-N=C=S]^–^ (Fig. S3).

Regarding indole GSL, 4-hydroxy glucobrassicin (ID 12), glucobrassicin (ID 13), and neoglucobrassicin (ID 15) were identified in the extracts (Table S1). They exhibited the deprotonated molecular ions [M-H]^−^ at *m/z* 463.0495, 447.0545, and 477.0652, respectively. MS-MS spectrum of glucobrassicin (ID 13) showed characteristic fragments at *m/z* 205 [M-Glc-SO_3_]^−^ and 252 [M-SGlc]^−^. The molecular ions of IDs 15 and 12 were 30 and 16 *m/z* units higher than that of peak 13, denoting the presence of an extra methoxy and a hydroxy group, respectively. Therefore, they were identified as neoglucobrassicin and 4-hydroxyglucobrassicin.

#### Isothiocyanates

Four isothiocyanates were identified in the first four minutes of the chromatographic run (Table S1 and Fig. S1). IDs 16, 17, 18, and 19 were assigned to methiin, erucin, sulforaphane, and 1-isothiocyanato-7-(methylsulfinyl) heptane, respectively. Methiin was previously reported in *B. oleracea* var. *capitata* in cauliflower, mustard, broccoli, and turnip [42, 43]. Erucin and sulforaphane were found in broccoli [44], while 1-isothiocyanato-7-(methylsulfinyl)heptane was present in cabbage, cauliflower, kale, and broccoli [45, 46].

#### Phenolic acids

Twenty-six phenolic acids and their derivatives were identified in the different extracts (from ID 20 to 45, Table S1 and Fig. S1). For instance, IDs 20, 21, 24, 27, 28, and 39 were simple phenolic acids assigned to salicylic, cinnamic, caffeic, ferulic, chlorogenic, and sinapic acids, respectively. Ferulic acid (ID 27, *m/z* 193.0507) was present as glycoside in IDs 31, 33, 34, and 41, as well as conjugated with sinapic acid glycosides in IDs 35, 36, 43, and 45. The MS-MS spectra of ferulic acid and some of its derivatives were characterized by the presence of fragment ions at *m/z* 178 and 134 indicating the sequential loss of methyl and CO_2_ groups. Similarly, p-coumaric acid glucoside (ID 26) exhibited a deprotonated molecular ion at *m/z* 325.0926 and fragment ions at *m/z* 163 and 119 due to the successive loss of a glucose moiety and a CO_2_ group [20]. Moreover, chlorogenic acid (ID 28) (Fig. S4) showed a deprotonated molecular ion peak at *m/z* 353.0880 and fragment ions indicating the quinic acid and caffeic acid moieties at *m/z* 191 and 179, respectively, as well as quinic acid and caffeic acid after loss of a CO_2_ group at *m/z* 161 and 135, respectively [47].

Several hydroxycinnamoyl gentiobioses have been previously reported in brassica vegetables [48]. For example, sinapoyl hydroxy feruloyl gentiobioside (ID 36, Fig. S5) exhibited a molecular ion at *m/z* 739.2081 and a fragment ion at *m/z* 415 due to the loss of a gentiobiose moiety [(M-H)-324]^–^. It also showed fragment ions due to sinapic acid fragmentation (*m/z* 223 [sinapic acid-H]^–^, 205 [sinapic acid-H-H_2_O]^–^, and 164 [sinapic acid-H-CH_3_-CO_2_]^–^), as well as hydroxy ferulic acid fragmentation (*m/z* 209 and 191). Regarding IDs 43 and 45, the parent ions were observed at *m/z* 723.2129 and *m/z* 929.2733, respectively. Both showed a fragment ion due to the loss of a dihexose unit from the parent ion (i.e., 399 and 605 *m/z*, respectively), besides characteristic fragment ions of sinapic acid and ferulic acid (*m/z* 193 [ferulic acid-H]^–^, 175 [ferulic acid-H-H_2_O]^–^). Therefore, they were assigned as feruloyl sinapoyl gentiobioside (ID 43, Fig. S6), and feruloyl disinapoyl gentiobioside, respectively (ID 45).

#### Flavonoids

A total number of 22 flavonoids, from ID 46 to ID 67, were identified in the t_r_ range between 6.5 and 9 min of the chromatographic run in the different extracts (Table S1 and Fig. S1). In agreement with the reported data [8, 10, 22, 48, 49], flavonols were the main flavonoids found in the extracts, among them 13 kaempferol, 6 quercetin, and 2 isorhamnetin derivatives, along with one flavanone (hesperetin glucoside). They were mostly present as mono-, di-, tri-, tetra-, and penta-*O*-glycosides limited mostly to glucosides and few rhamnosides [50]. *O*-acylated flavonols with hydroxycinnamic acids (caffeic, sinapic, and ferulic acids) were also identified. The sugar types could be identified by the sequential elimination of pentosyl (-132 *m/z*), rhamnosyl (-146 *m/z*), and hexosyl (-162 *m/z*) moieties. The identified flavonol glycosides were mainly of sophoroside type, as previously reported for the genus *Brassica*, which is characterized by the fragment ions corresponding to (-120 *m/z*) and (-180 *m/z*). Twelve non-acylated glycosides were identified among them six kaempferol glycosides in IDs 47, 49, 54, 63, 64, and 66. Fig. S7 shows the MS/MS spectrum of kaempferol-3-*O*-rhamnosyl-7-*O*-hexoside (ID 63) with [M-H]^–^ at *m/z* 593.1509 and fragment ions at *m/z* 446 resulting from the elimination of rhamnosyl radical *via* hemolytic cleavage of the 3-*O*-glycosidic bond and at *m/z* 431 generated by the loss of a hexose at 7-position (-162 *m/z*). Four quercetin glycosides were identified in IDs 48, 50, 53, and 65. While two isorhamnetin glycosides were in IDs 51 and 60. Fig. S8 shows the MS/MS spectrum of brassicoside (ID 51), which showed [M-H]^–^ at *m/z* 801.2087 *m/z*, and fragment ions at *m/z* 639, 476, and 315 indicating the successive loss of three glucosyl units, with the final fragment denoting the isorhamnetin aglycone ion.

Moreover, seven acylated kaempferol glycosides were identified as shown in IDs 56, 57, 58, 59, 61, 62, and 67, as well as one acylated quercetin glucoside in ID 55 that was assigned to quercetin 3-*O*-(2-feruloyl sophoroside)-7-*O*-glucoside. The MS/MS spectrum of this metabolite (ID 55, Fig. S9) showed the molecular ion [M − H]^−^ at *m/z* 963.2396 and three major fragment ions, at *m/z* 787 [M-H-feruloyl]^−^, 625 [M-H-feruloyl-Glc]^−^, and 462 [M-H-feruloyl-2Glc]^−^ indicated the successive loss of one feruloyl moiety followed by two glucosyl residues. While, kaempferol 3-(2-caffeoyl sophoroside)-7-*O*-glucoside (ID 56, Fig. S10) displayed [M-H]^−^ at *m/z* 933.2307 and fragment ions due to successive loss of glucosyl residues, together with fragment ions at *m/z* 179 and 161 due to caffeic acid and caffeoyl radical, respectively [8, 48]. A similar fragmentation pattern was also found in kaempferol-3-*O*-sinapoyl sophorotrioside-7-*O*-glucoside (ID 57, Fig. S11), that exhibited fragment ions due to successive loss of glucosyl moieties (-162 *m/z* at *m/z* 977), sinapoyl (-206 *m/z* at *m/z* 771), and at *m/z* 447 corresponded to the loss of sinapoyl and 3 glucosyl moieties, as well as those produced by the inter-glycosidic bond of the sophorotrioside moiety fragmentation at *m/z* 753 and 591 or the characteristic ions of sinapic acid.

#### Organic and fatty acids

Seven organic acids were identified as fumaric (ID 68), malic (ID 69), citric (ID 70), succinic (ID 71), methyl citric (ID 72), quinic (ID 73), and shikimic acids (ID 75) (Table S1 and Fig. S1) and were previously reported in *B. oleracea* L. var. *costata* [49]. Most of the fatty acids were eluted from 8 to 20 min of the chromatographic run. In agreement with the literature [51], a total of 15 long chain mono-, di-, tri-, and polyunsaturated fatty acids (IDs 74, 76–99) were identified. For example, the [M-H]^−^ ions of IDs 89 and 90 differed in 2 *m/z* units (295.2276 vs. 297.2433 *m/z*), hence denoting an extra double bond, as found in hydroxy octadecadienoic acid (ID 89) compared to hydroxy oleic acid (ID 90).

### MIC and EIC of the extracts against *A. baumannii* and the effect of sub-inhibitory concentrations on growth curves

The antibacterial activity of the six leaf extracts was evaluated measuring MIC against *A. baumannii*. The six extracts failed to inhibit the growth of *A. baumannii* at the highest tested concentration (512 µg/mL) indicating that they did not have notable direct antibacterial activity against this strain at the tested concentration.

To determine the effective killing concentrations of the six extracts, the TTC reduction assay was used (Table 1). RP and GP showed the highest killing activity by having the lowest effective killing concentration (i.e., 520 µg/mL). In contrast, BR, TK, and CC showed the lowest activity by not being able to kill *A. baumannii* until a concentration of 640 µg/mL. Finally, CK had an intermediate value of 560 µg/mL (Table 1).

**Fig. 1:**
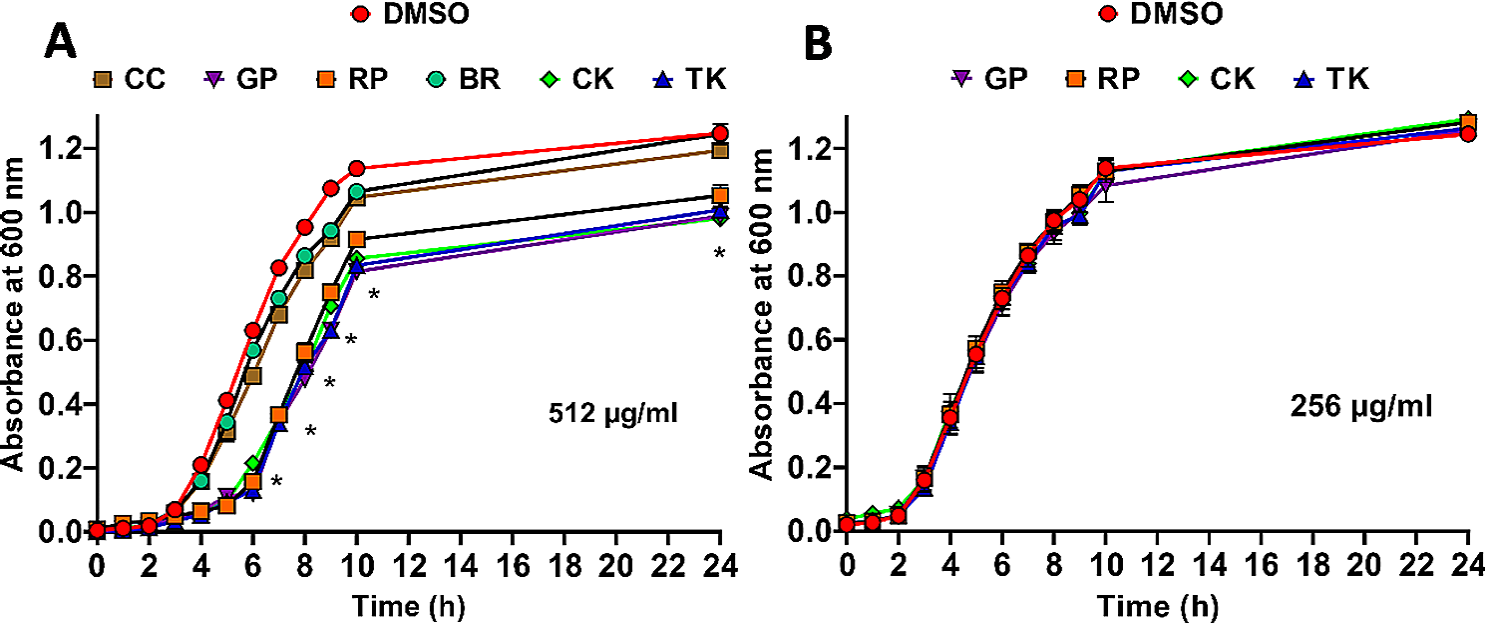
Growth curves analyses of *A. baumannii* in the presence of the *Brassica* leaf extracts. * Indicates that the difference is statistically significant as determined by the student t-test (*p* < 0.05, *n* = 3). Chinese cabbage (CC), Curly kale (CK), Tuscan kale (TK), red Pak choi (RP), green Pak choi (GP), and Brussels sprouts (BR)

Since we could not determine a MIC value below 512 µg/mL for all six extracts, we wanted to test if they would have an impact on the growth pattern of *A. baumannii*. At 512 µg/ml, only BR and CC showed no significant effect on the growth curve of *A. baumannii* (Fig. 1A), because their curves almost overlapped with the control DMSO curve. In contrast, at this concentration all the other four extracts showed a significant delay in the time point at which the logarithmic phase started and a negative impact on the maximum extent of growth that could be reached (Fig. 1A). To confirm that this effect was only observed at high concentration, growth curve analyses of these four extracts were repeated at a lower concentration (256 µg/mL), and no impact on the growth curves was observed (Fig. 1B). It is worth mentioning, that in the case of the drug molecule orlistat, the concentration that showed no effect on bacterial growth was 64 µg/mL (data not shown). Therefore, despite the six extracts presenting negligible direct killing activity against *A. baumannii*, at least four of them had some effect on bacterial growth at a high concentration.

**Table 1:** Effective killing concentrations of the *Brassica* leaf extracts

Species	Effective killing concentration (µg/ml)	Concentration used in the lipase assay (µg/ml)	
Curly kale (CK)	560	256	
Red pak choi (RP)	520	256	
Green pak choi (GP)	520	256	
Tuscan kale (TK)	640	256	
Chinese cabbage (CC)	640	512	
Brussels sprouts (BR)	640	512	

SD: standard deviation

### Inhibitory effects against *A. baumannii* lipases

*A. baumannii* has many virulence factors, including extracellular components with hemolytic, phospholipase, protease, and iron-chelating activities, biofilm formation, surface motility, and stress resistance, which enhance its bacterial toxicity and pathogenicity [52]. While research has focused on understanding the mechanisms of antibacterial resistance, biofilm formation, and epidemiology, little is known concerning the role of secreted proteins in *A. baumannii* survival and propagation during infection. Targeting virulence factors with anti-virulence agents may help to prevent the development of resistance to antibiotics [53].

**Fig. 2:**
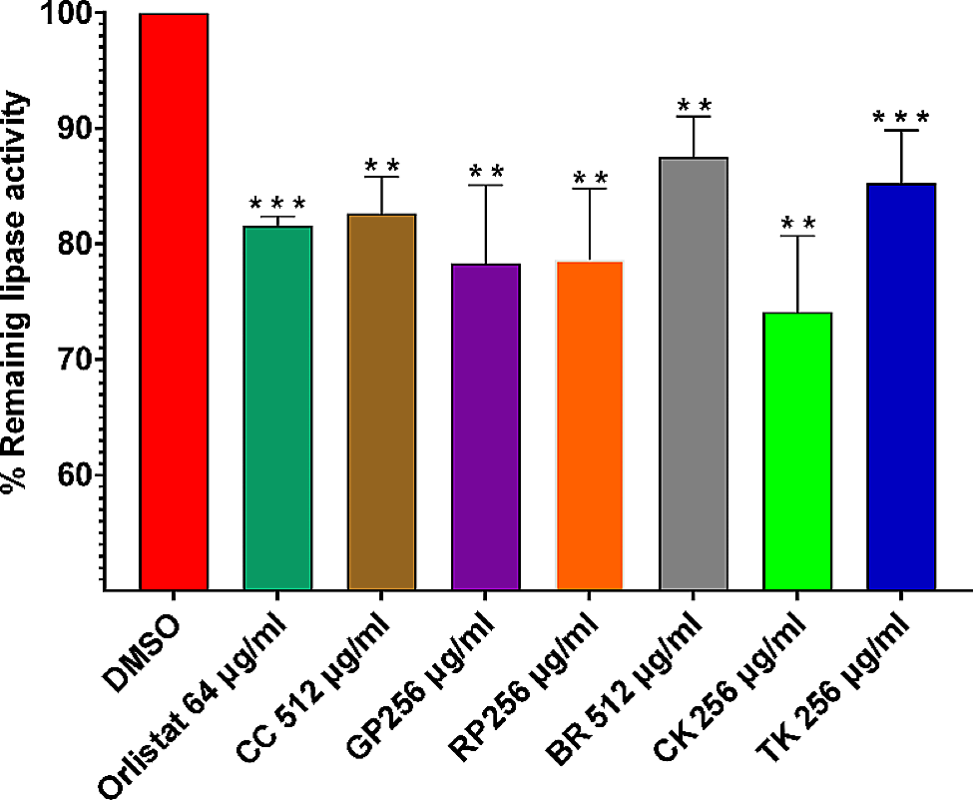
Remaining *A. baumannii* lipolytic activity after treatment with the *Brassica* leaf extracts for 3 h. Error bars are standard deviations of the mean, and ** and *** indicate that the *p*-value is < 0.01 and < 0.001 as determined by the student t-test (*n* = 3), respectively. Chinese cabbage (CC), Curly kale (CK), Tuscan kale (TK), red Pak choi (RP), green Pak choi (GP), Brussels sprouts (BR)

Lipases and phospholipases are important virulence factors of *A. baumannii* [52]. These enzymes degrade lipids, which are essential components of cell membranes, to facilitate nutrient acquisition, adhesion, colonization, multiplication, and invasion [54]. Lipases hydrolyze a wide range of esters, and esterolytic activity is routinely estimated by employing the pNPP assay [55]. The six extracts significantly inhibited the lipolytic activity of *A. baumannii* to varying extents, as shown in Fig. 2. Notably, these extract concentrations did not affect the bacterial growth itself (Fig. 1).

The highest activity was observed with CK with ∼ 26% inhibition at 256 µg/mL concentration, followed by both RP and GP with ∼ 21%, and TK with ∼ 15%. In contrast, BR and CC demonstrated the lowest anti-lipolytic activity with only ∼ 12.5% and ∼ 17.5% inhibition, respectively at 512 µg/mL concentration. It is worth mentioning that orlistat was shown before to bind specifically to a lipase of another species of *Acinetobacter* (i.e. *A. radioresistens*) [56]. In our hands, the orlistat inhibited the lipolytic activity by ∼ 20% at a concentration of 64 µg/mL. These findings indicated that the extracts of the investigated *Brassica* species would present *A. baumannii* lipase inhibitors, hence that they would have beneficial therapeutic activity against the infections caused by this notorious pathogen.

### Multivariate data analysis

To have a better understanding of the differential metabolite distribution among the six *Brassica* leaf extracts and correlate the identified metabolites with the observed effects on bacterial growth and lipolytic inhibition, the complete set of 3697 compounds detected in the LC-MS metabolic profiles of the different species were subjected to unsupervised HCA and PCA, followed by supervised OPLS-DA [57].

#### Unsupervised HCA and PCA

**Fig. 3:**
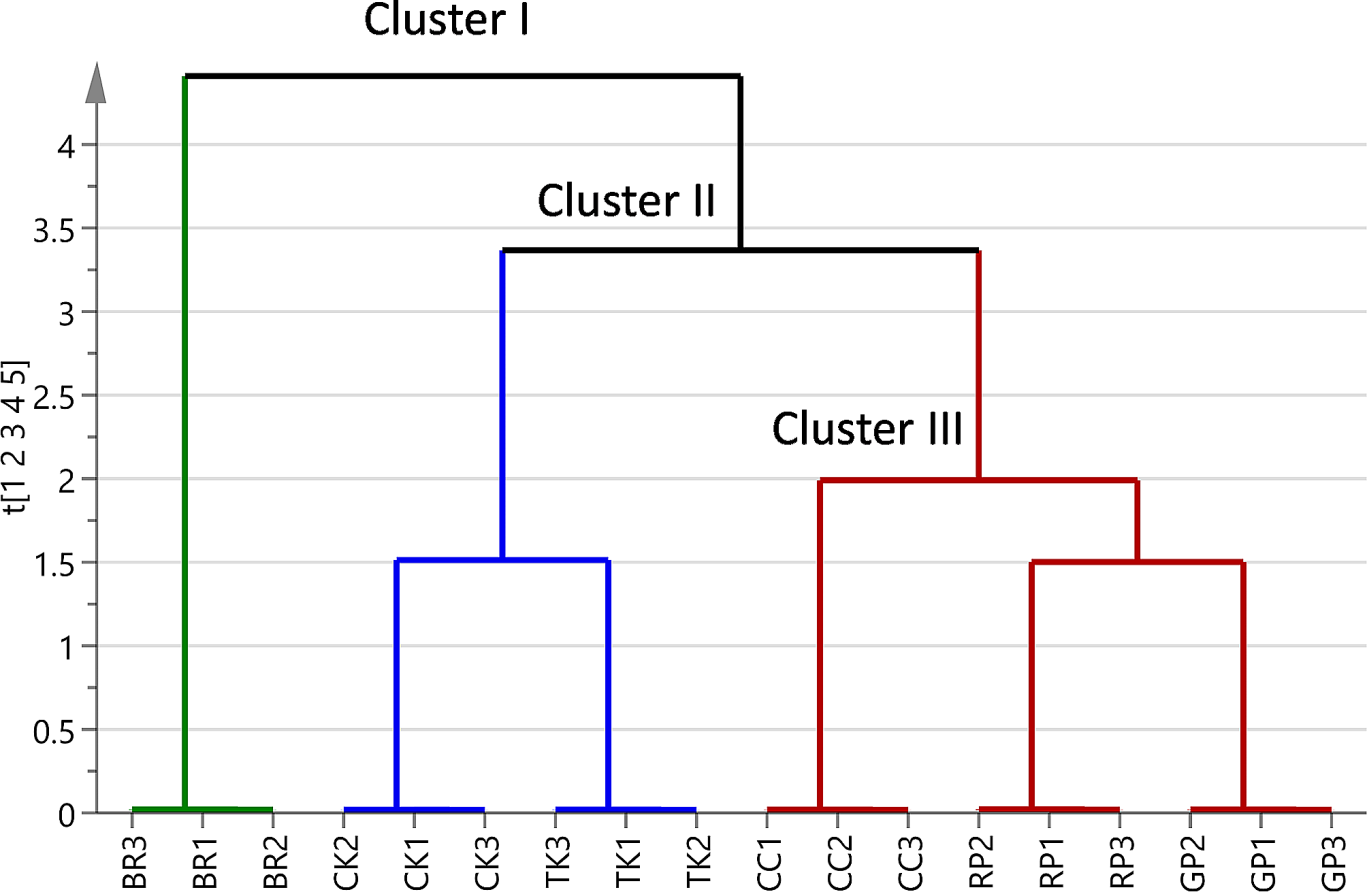
Hierarchical cluster analysis (HCA) of the *Brassica* leaf extracts based on cluster analysis of LC-MS profiles. Chinese cabbage (CC), Curly kale (CK), Tuscan kale (TK), red Pak choi (RP), green Pak choi (GP), Brussels sprouts (BR)

HCA is an unsupervised multivariate data analysis method to explore sample heterogeneity in an intuitive graphical display called a dendrogram. HCA is an excellent tool for preliminary data analysis, where similar samples are grouped forming clusters and the distance between clusters is related to their similarity degree [58]. The HCA dendrogram (Fig. 3) shows that the six extracts were grouped into three clusters (I to III) according to their metabolic profiles. Cluster I comprised only BR and the great distance from clusters II and III, which grouped CK and TK and CC, RP, and GP, respectively, suggested important composition differences. Although HCA allows a rapid and simple preliminary data analysis, it is more informative to examine the dendrogram in conjunction with PCA.

**Fig. 4:**
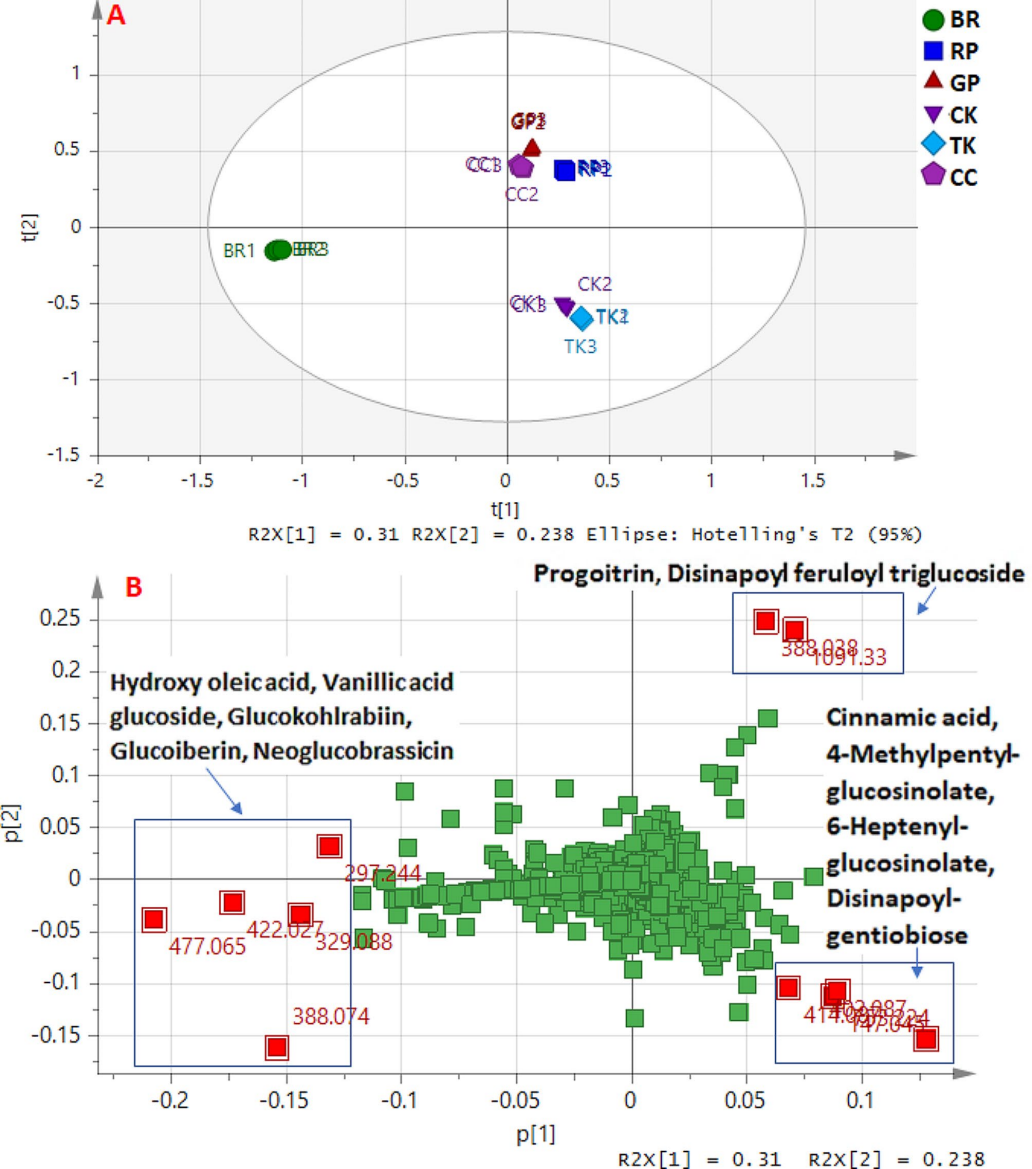
Principal Component Analysis (PCA) of the *Brassica* leaf extracts. (**A**) Scores plot and (**B**) loading plot. The identified metabolites showing the largest absolute score values along each PC are named and colored in red and denoted by *m/z* and name. Chinese cabbage (CC), Curly kale (CK), Tuscan kale (TK), red Pak choi (RP), green Pak choi (GP), Brussels sprouts (BR)

PCA can be used to find trends and classify the different leaf extracts into different groups according to their metabolic profiles [58]. In the constructed PCA model (Fig. 4), clusters from the different extracts were located at distinct positions of the PCA score plot of the two principal components, which accounted for 31.0% (PC1) and 23.8% (PC2) of the explained variance, and no outliers were observed beyond the Hotelling’s T2 ellipse that included the 95% confidence area (Fig. 4A). In the PCA score plot (Fig. 4A), BR was clearly separated from the rest of the species along PC1, as well as along PC2 were observed three groups corresponding to CK and TK, BR and the rest of species (GP, RP, and CC). Such clustering and segregation in the PCA indicated possible chemical similarities and differences among the metabolic profiles of the studied species. Species discrimination in PCA was explained in terms of the detected compounds using the loading plot (Fig. 4B), which showed the compound contribution to the PC scores. The compounds with the largest absolute score values along each PC, which are colored in red and denoted by name in the PCA loading plot, if metabolite identity was available (Fig. 4B), were considered the most relevant to explain the three groups observed in the PCA score plot. Hydroxy oleic acid (ID 90), vanillic acid glucoside (ID 29), glucokohlrabiin (ID 4), glucoiberin (ID 2), and neoglucobrassicin (ID 15) were found to contribute the most to discriminate BR from the other species. While progoitrin (ID 3) and disinapoyl feruloyl triglucoside (ID 37) were predominated mainly in CC, RP, and GP, as well as cinnamic acid (ID 21), 4-methylpentyl glucosinolate (ID 8), 6-heptenyl glucosinolate (ID 10), and disinapoyl gentiobiose (ID 42) in TK and CK. As can be observed, glucosinolates and phenolic compounds were the main metabolites responsible for species discrimination.

#### Supervised OPLS-DA for metabolites-bioactivity correlation

**Fig. 5:**
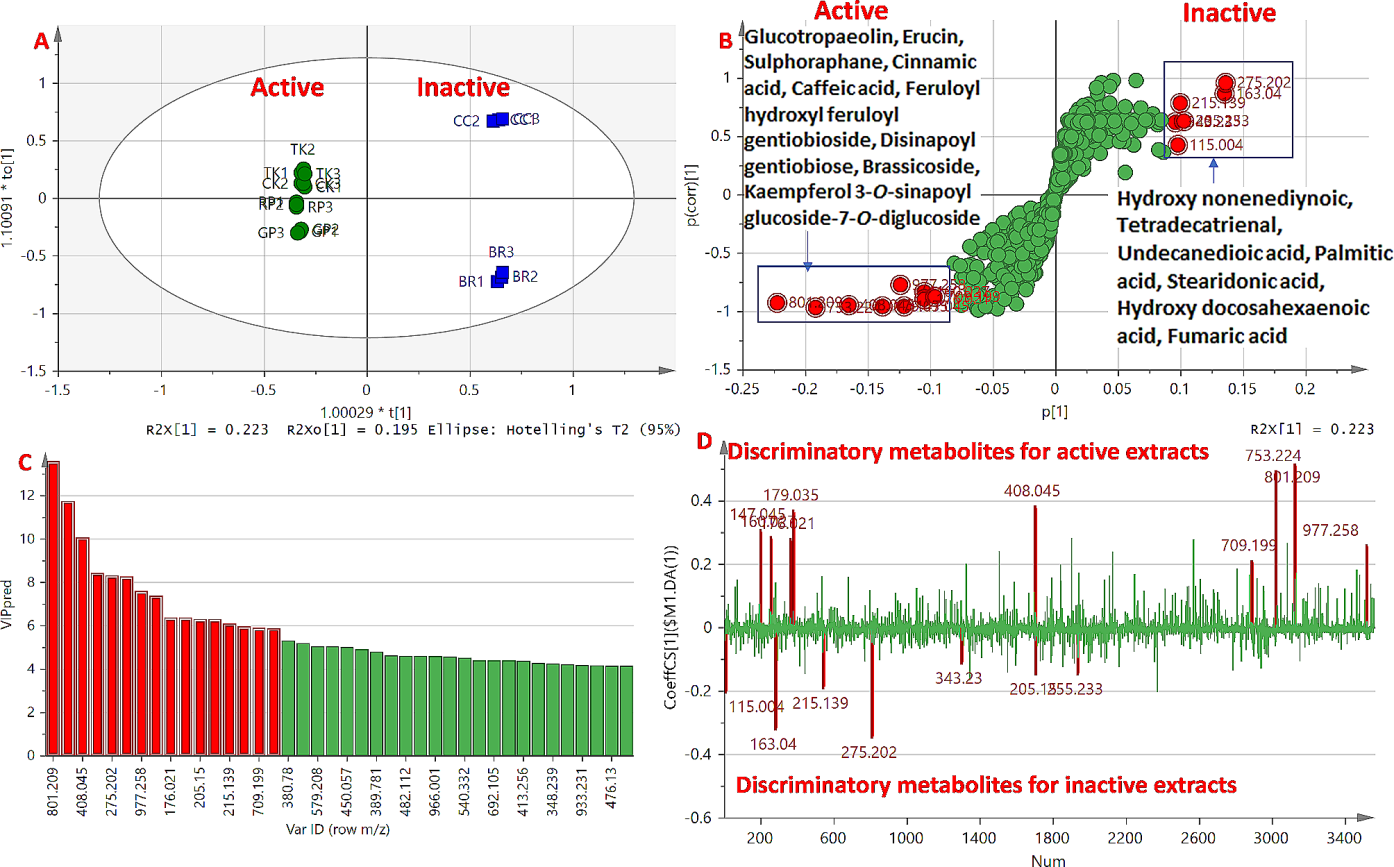
Orthogonal Projection on Latent Structure-Discriminant Analysis (OPLS-DA) to investigate the growth inhibitory and lipolytic effects of the *Brassica* leaf extracts. A two-class OPLS-DA model was constructed with the four active extracts (TK, CK, RP, and GP) against the inactive ones (CC and BR). (**A**) score plot, (**B**) loading S-plot, (**C**) zoomed VIP score plot, and (**D**) zoomed correlation coefficient plot, (Compounds contributing the most to class discrimination were colored in red and denoted by name in (**B**), and listed in detail in Table 2)

OPLS-DA is a supervised multivariate data analysis method that can be applied to find potential specific correlations between the identified metabolites and the bioactivity results, by classifying the leaf extract samples into two groups, e.g., bioactive and non-bioactive, hence only the metabolites related to the bioactivity are influencing the groups discrimination [44, 58]. In this study, four extracts, TK, CK, RP, and GP, demonstrated significant effects on bacterial growth of *A. baumannii* and lipolytic activity inhibition. Therefore, a two-class OPLS-DA model was constructed with the four active extracts against the inactive extracts (CC and BR). The OPLS-DA score plot (Fig. 5A) explained 99% of the total variance (R2(X) = 0.547, R2(Y) = 0.999) with a prediction goodness parameter Q2 = 0.97 at *p* < 0.05. For model validation, the receiver operating characteristic (ROC) curve was calculated and the area under the curve was found to be 1.0, indicating the effectiveness of the classification model (Fig. S12A). A permutation test (20 iterations) and CV-ANOVA were conducted to evaluate whether the model was overfitted (Fig. S12B-C), with a negative Q2 intercept value and *p*-value < 0.05 indicating the model validity. The root mean square error of estimation (RMSEE) was 0.018 and the root mean square error of cross-validation (RMSECV) was 0.081, indicating the accuracy and the good prediction power of the model [20]. The main contributing metabolites to the bioactivities were identified in the loadings S-plot, which compared the variable magnitude against its reliability. These metabolites were further confirmed using the variable importance in the projection (VIP) and the correlation coefficient plots (Fig. 5C-D) and they are listed in detail in Table 2. As a result, nine discriminating metabolites responsible for the growth inhibitory and lipolytic effects of the active extracts (TK, CK, RP, and GP) were identified, with VIP scores > 5 and positive coefficient values (colored in red in Fig. 5C-D and listed with details in Table 2). They included one glucosinolate (ID 5), two isothiocyanates (IDs 17 and 18), four phenolic acids (IDs 21, 24, 41, and 42), and two flavonoids (IDs 51 and 59). In contrast, one organic acid (ID 68) and six fatty acids (IDs 74, 76, 79, 85, 88, and 91) were the most relevant metabolites to discriminate the inactive extracts (CC and BR) with VIP scores > 5 and negative correlation coefficients (Table 2). It is worth noting that numerous phenolic acids and flavonoids were reported to exhibit antibacterial activity against *A. baumannii* [59]. Several kaempferol and isorhamnetin glycosides were reported to present antibacterial and anti-virulence activities through different mechanisms, such as inhibition of sortase enzyme, biofilm formation, reduction of bacterial adhesion, and invasion [60]. It was previously reported that cinnamic and benzoic derivatives of phenolic acids (caffeic acid, gallic acid, and protocatechuic acid) are effective prooxidants with low redox potential due to their catechol rings and hydroxyl groups, thereby increasing bacterial death by triggering redox imbalance in the cells, resulting in damage to cellular macromolecules such as carbohydrates, proteins, lipids, and DNA [59]. Additionally, combining cinnamic acid with various antibiotics inhibited the expression of the biofilm-associated genes against *A. baumannii* [61]. Interestingly, sulforaphane and erucin were described to have antibacterial and anti-virulence activity against *Pseudomonas aeruginosa*, and now we found that they may possess a similar inhibition against *A. baumannii* [62]. Moreover, glucosinolates demonstrated antibacterial activity against a wide range of Gram-positive and Gram-negative bacteria, in addition to exerting a synergetic antibacterial effect against *A. baumannii* when combined with common antibiotics [63].

**Table 2:** VIP scores and correlation coefficients of the metabolites positively and negatively correlated to the growth inhibitory and lipolytic activities of the six *Brassica* leaf extracts as obtained from OPLS-DA

Metabolite	VIP scores	Correlation coefficients
**Positive correlation**		
Isorhamnetin 3-sophoroside 7-glucoside (Brassicoside) (ID 51)	13.6	0.52
Disinapoyl gentiobiose (ID 42)	11.8	0.50
Glucotropaeolin (ID 5)	10.1	0.39
Caffeic acid (ID 24)	8.4	0.37
Kaempferol 3-*O*-sinapoyl glucoside-7-*O*-diglucoside (ID 59)	7.6	0.26
Cinnamic acid (ID 21)	7.4	0.31
Sulforaphane (ID 18)	6.4	0.29
4-(Methylthio)butyl mustard oil (Erucin) (ID 17)	6.3	0.27
Feruloyl hydroxyl feruloyl gentiobioside (ID 41)	5.9	0.21
**Negative correlation**		
Hydroxy nonenediynoic (ID 76)	8.3	-0.32
Stearidonic acid (ID 88)	8.3	-0.35
Fumaric acid (ID 68)	6.2	-0.21
Undecanedioic acid (ID 74)	6.1	-0.19
Tetradecatrienal (ID 91)	6.3	-0.15
Palmitic acid (ID 79)	6.3	-0.15
Hydroxy docosahexaenoic acid (ID 85)	5.9	-0.12

### Molecular docking

Selected biomarkers, representatives from each class of positively correlated metabolites, including a flavonoid (brassicoside (ID 51)), a glucosinolate (glucotropaeolin (ID 5)), two phenolic acids (caffeic acid (ID 24) and cinnamic acid (ID 21)), and two isothiocyanates (sulforaphane (ID 18) and erucin (ID 17)), were subjected to molecular docking to investigate the putative interaction mechanism and predict the possible binding modes with crystallized structure of *A. baumannii* lipase binding site. As summarized in Table 3, all the selected metabolites exhibited interactions with lipase, displaying binding energies ranging from −70.25 to −30.20 kcal/mol. Brassicoside (ID 51) and glucotropaeolin (ID 5) presented higher binding energies compared to the reference binding ligand (i.e. orlistat, -50.13 kcal/mol). Various secondary interactions, such as hydrogen bonding, hydrophobic, and aromatic stacking interactions, were raised between the selected metabolites and different amino acid residues of the crystallized structure of the *A. baumannii* lipase receptor. These interactions likely contributed to the observed inhibitory effect of the selected metabolites.

**Table 3:** Summary of the free binding energy (∆G), hydrogen bonding interactions, aromatic stacking, and hydrophobic interactions of the selected metabolites and orlistat with the crystallized structure of the *A. baumannii* lipase receptor

Compound	∆G / (kcal / mol)	Hydrogen bonding interactions	Aromatic stacking interactions	Hydrophobic interactions
Brassicoside (ID **51**)	-70.25	Ala126, Trp220, Trp222 Ser257, Ser258, and Arg260	Phe111, Trp114, and Trp220	Leu110, Leu129, and Leu167
Glucotropaeolin (ID **5**)	-60.85	Glu113, Lys125, Ser127, Ser218, and Arg260	Phe111, Trp114, Trp220, and Trp222	Ala79, Leu129, and Leu167
Caffeic acid (ID **24**)	-50.97	Ser257	Phe111, Trp114, Trp220, and Trp222	Ala79, Leu110, Leu129, and Leu167
Cinnamic acid (ID **21**)	-40.99	Ala126	Phe111, Trp114, Trp220, and Trp222	Ala79, Leu110, and Leu167
Sulforaphane (ID **18**)	-40.20	Arg260	Phe111, Trp114, Trp220, and Trp222	Leu110
Erucin (ID **17**)	-30.20	No	Phe111, Trp114, Trp220, and Trp222	Leu110
Orlistat	-50.13	Ala126, Ser257, and Arg260		Ala79, Leu110, Phe111, Trp114, Leu129, Trp166, Leu167, Trp220, and Trp222

**Fig. 6:**
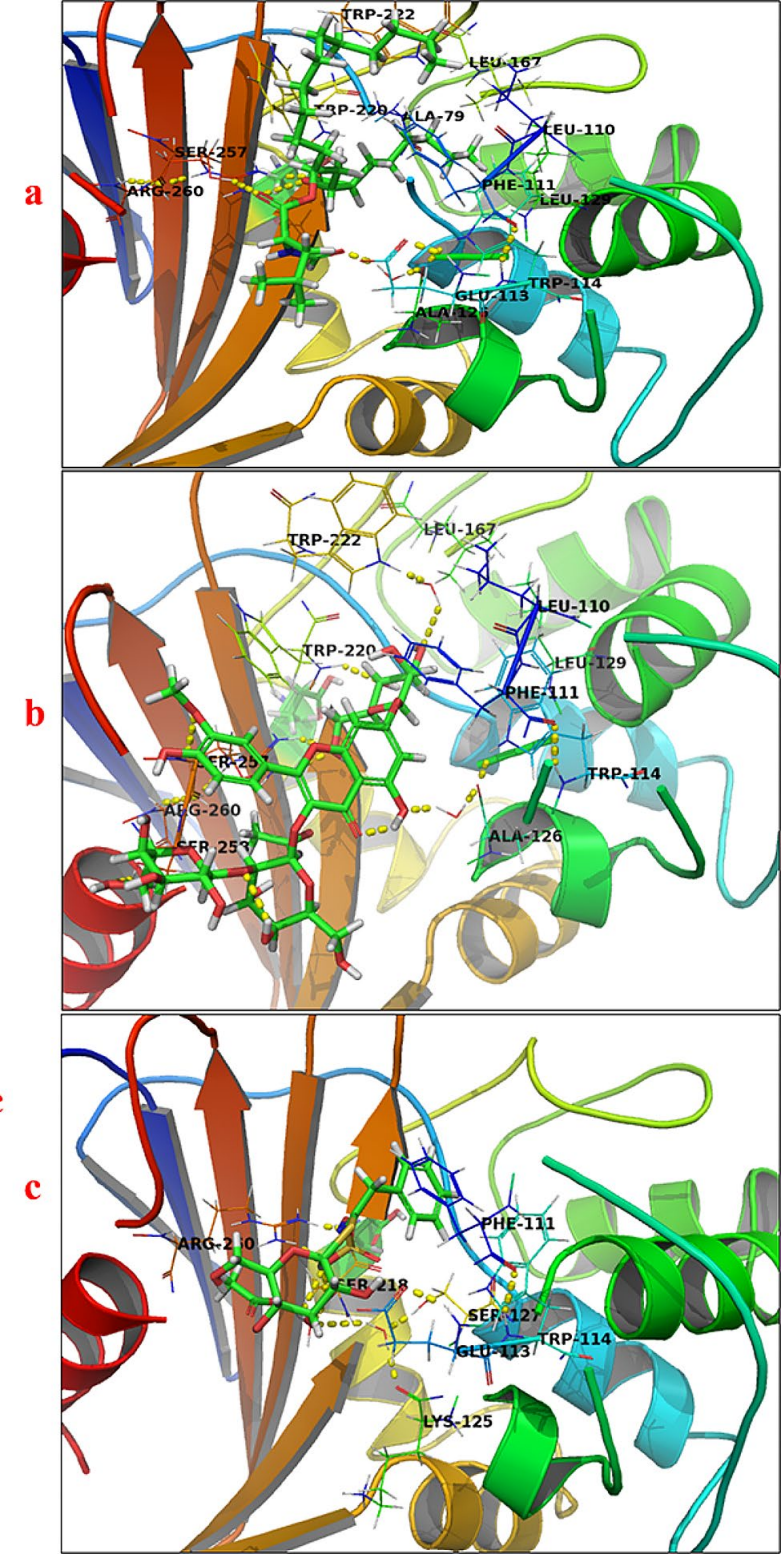
Crystal structure of *A. baumannii* lipase receptor with **a**: orlistat.; **b**: brassicoside (ID 51); **c**: glucotropaeolin (ID 5); The hydrogen bonding interactions are presented by dashed lines. Residues contacting ligands are demonstrated as lines

The predicted binding mode for orlistat displayed that both oxygen atoms and the attached carbonyl group at the 2-position of oxetane were involved in a hydrogen bonding interaction with Arg260. Furthermore, the carbonyl group of formamide moiety participated in a water-mediated interaction with the backbone carbonyl group of Ala126. Additionally, the carbonyl group of orlistat formed another hydrogen bond with Ser257. In terms of hydrophobic interactions, the hexyl moiety of orlistat predominantly occupied the hydrophobic pocket formed by Ala79, Trp114, Leu129, Trp166, and Leu167, while the tridecane showed hydrophobic interactions with Leu110, Phe111, Trp220, and Trp222. In contrast, the isopentane moiety was engaged in an unfavorable interaction with Glu113. Consequently, hydrophilic residues played essential roles in the binding of orlistat to the crystallized structure of *A. baumannii* lipase receptor (Fig. 6a).

The predicted binding mode for brassicoside (ID 51) revealed that its polyphenolic moieties were oriented to the enzyme active site, engaging in hydrogen bonding interactions with the backbone amino groups of Trp220, Ser257, Ser258, and Arg260. Additionally, the phenolic groups participated in hydrogen bonding interactions with the backbone carbonyl groups of Ala126, Trp220, and Trp222, along with aromatic stacking interactions with Phe111, Trp114, and Trp220. Additionally, these groups were involved in hydrophobic interactions primarily with Leu110, Leu129, and Leu167. Figure 6b presents the docked metabolite inside the binding pocket. The predicted binding mode for glucotropaeolin (ID 5) suggested that the dihydroxy groups of the pyran moiety were involved in hydrogen bonding and water-mediated interactions with Glu113. In addition, the benzyl moiety was stabilized through aromatic stacking interactions with Phe111, Trp114, Trp220, and Trp222, and was located in the hydrophobic pocket formed by Ala79, Leu129, and Leu167. On the other hand, the sulfonic moiety was surrounded by hydrogen bonding interactions with Arg260, and water-mediated interactions with Lys125, Ser127, and Ser218, which formed a pocket that contributed to an enhanced affinity of glucotropaeolin with the binding site (Fig. 6c). Furthermore, the molecular docking result for caffeic acid (ID 24) demonstrated that the carboxylic moiety was anchored by a hydrogen bonding interaction with the gatekeeper residue, Ser257. Additionally, the styrene moiety was located in hydrophobic interactions with Ala79, Leu110, Leu129, and Leu167, and formed aromatic stacking interactions with Phe111, Trp114, Trp220, and Trp222. Finally, its hydroxyl group was located at a distance of 2.7 Å from Ser80 (Table 3, Fig. S13a). On the other hand, the binding mode of cinnamic acid (ID 21) was similar to that of caffeic acid (ID 24) with an affinity value of -40.99 kcal/mol. In this case, its carboxylic and styrene moieties maintained the same hydrogen bonding, hydrophobic, and aromatic stacking interactions. However, the favorable interaction of hydroxyl moiety with Ser80 was abolished, which may explain its lower affinity compared with caffeic acid (ID 24) (Table 3, Fig. S13b).

Additionally, the proposed binding mode of sulforaphane (ID 18) with the crystal structure of *A. baumannii* lipase indicated that the sulfinyl group formed hydrogen bonding interactions with Arg260, while the butane moiety was involved in hydrophobic interactions with Leu110, Phe111, Trp114, Trp220, and Trp222. The binding mode of erucin (ID 17) was similar to sulforaphane however, the hydrogen bonding interaction with Arg260 was annulled (Table 3, Fig. S13c). Overall, an examination of the interacting amino acid residues in Table 3 revealed consistent binding modes among all selected metabolites, with brassicoside (ID 51) and glucotropaeolin (ID 5) exhibiting more extensive interactions across all three types of interactions, similar to orlistat. Upon analyzing the favorable binding poses, four essential hydrogen bonding interactions were identified, involving Glu113, Ser257, Ser258, and Arg260 residues. Additionally, the contributions of aromatic stacking resulted from a more deeply located binding pose within the binding site of the *A. baumannii* receptor. These hydrogen bonding interactions, along with aromatic stacking interactions, particularly with aromatic amino acid residues Phe111, Trp114, Trp220, and Trp222, appeared to be pivotal factors contributing to the higher affinity of brassicoside and glucotropaeolin towards the *A. baumannii* receptor, playing a significant role in bacterial lipase inhibition.

## Conclusions

In conclusion, our results are promising and support the use of *Brassica* leaf extracts as novel sources of natural antibacterial and anti-virulence agents for multi-drug resistant bacteria. Notably, extracts from TK, CK, RP, and GP exhibited the most substantial profiles of bioactive metabolites. Among these bioactive metabolites, the flavonoid brassicoside and the glucosinolate glucotropaeolin displayed the highest potential for inhibiting the *A. baumannii* lipase enzyme. This discovery holds significance not only for pharmaceutical applications but also for the development of novel breeding programs focused on *Brassica* vegetables as functional foods and valuable sources of nutraceuticals.

### Electronic supplementary material

Below is the link to the electronic supplementary material.

Supplementary Material 1

## Data Availability

The data supporting the findings of this study are available within the article and its supplementary file.

## Abbreviations

CV-ANOVA: cross-validated analysis of variance
HCA: Hierarchical cluster analysis
LC-QTOF-MS/MS: liquid chromatography-quadrupole time-of-flight tandem mass spectrometry
PCA: principal component analysis
RMSEcv: root mean square of error of cross-validation
RMSEE: root mean square error of estimation
ROC: receiver operating characteristic
VIP: variable importance to projection
