# Supplementary material for: Evidence on the inhibitory effect of Brassica plants against Acinetobacter baumannii lipases: phytochemical analysis, in vitro, and molecular docking studies
Source: BMC Complement Med Ther. 2024 Apr 19;24:164. doi: 10.1186/s12906-024-04460-y (PMC11027383; doi:10.1186/s12906-024-04460-y)
Supplement: Supplementary file 1 — Supplementary Material 1 [file 12906_2024_4460_MOESM1_ESM.pdf]

SUPPLEMENTARY FILE

Evidence on the inhibitory effect of *Brassica* plants against *Acinetobacter baumannii* lipases: phytochemical analysis, in vitro, and molecular docking studies

Manal M Sabry[1], Ali M El-Halawany[1], Walaa G. Fahmy[2], Basma M. Eltanany[3], Laura Pont[4,5], Fernando Benavente[4], Ahmed S. Attia[2,6], Farag F. Sherbiny[7], Rana M. Ibrahim[1]*

[1] Department of Pharmacognosy, Faculty of Pharmacy, Cairo University, Cairo 11562, Egypt. E-mail: manal.sabry@pharma.cu.edu.eg; ali.elhalawany@pharma.cu.edu.eg; rana.mohamed@pharma.cu.edu.eg

[2] Department of Microbiology & Immunology, Faculty of Pharmacy Cairo University, Cairo 11562, Egypt. E-mail: walaa.gamal@pharma.cu.edu.eg; ahmed.attia@pharma.cu.edu.eg

[3] Department of Analytical Chemistry, Faculty of Pharmacy, Cairo University, Cairo 11562, Egypt. E-mail: basma.el-tanany@pharma.cu.edu.eg

[4] Department of Chemical Engineering and Analytical Chemistry, Institute for Research on Nutrition and Food Safety (INSA·UB), University of Barcelona, Barcelona 08028, Spain. E-mail: laura.pont@ub.edu; fbenavente@ub.edu

[5] Serra Húnter Program, Generalitat de Catalunya, Barcelona 08007, Spain.

[6] School of Pharmacy, Newgiza University, Giza 12577, Egypt

[7] Pharmaceutical Organic Chemistry Department, Faculty of Pharmacy, Al-Azhar University, Nasr city 11884, Cairo, Egypt. E-mail: dr-farag-sherbiny@azhar.edu.eg

* Correspondence: rana.mohamed@pharma.cu.edu.eg

Table S1. Metabolites identified in the methanolic *Brassica* leaf extracts by LC-QTOF-MS/MS in negative ESI mode.

ID number	*t_r (min)	Detected Molecular ion $(M\text{-}H)^-$	*Error ppm	Molecular formula	Identification	**MS/MS (m/z)	Species[a]
				**Glucosinolates**			
1	1.03	358.0269	-0.8	$C_{10}H_{17}NO_9S_2$	Sinigrin	259, 200, 179, 164, 116, 96, 74	CC, CK, TK, RP, GP, BR
2	1.04	422.0272	4.1	$C_{11}H_{21}NO_{10}S_3$	Glucoiberin	406, 341, 274, 259, 245, 225, 195, 180, 165	CC, CK, TK, RP, GP, BR
3	1.07	388.0381	0.9	$C_{11}H_{19}NO_{10}S_2$	Progoitrin	340, 299, 259, 179, 135, 96	CC, RP, GP
4	1.36	388.0743	0.5	$C_{12}H_{23}NO_9S_2$	Glucokohlrabiin	351, 299, 224, 183, 131, 95	CC, CK, TK, RP, GP, BR
5	1.44	408.0450	5.3	$C_{14}H_{19}NO_9S_2$	Glucotropaeolin	275, 259, 241, 195, 166	CK, TK, RP, GP
6	1.55	450.0574	1.4	$C_{13}H_{25}NO_{10}S_3$	Glucoalyssin	386, 328, 259, 192, 165, 144, 128	RP, GP
7	1.59	402.0537	0.8	$C_{12}H_{21}NO_{10}S_2$	Gluconapoleiferin	259, 195, 161, 128, 96	RP
8	1.59	402.0874	5.3	$C_{13}H_{25}NO_9S_2$	4-Methylpentyl glucosinolate	392, 248, 209, 173, 149, 121, 84	CC, CK, TK, RP, GP, BR
9	1.77	372.0433	1.3	$C_{11}H_{19}NO_9S_2$	Gluconapin	322, 285, 259, 224, 178, 127, 96, 74	RP, GP, BR
10	2.06	414.0872	-6.2	$C_{14}H_{25}NO_9S_2$	6-Heptenyl glucosinolate	206, 172, 144, 96	CC, CK, TK, RP, GP, BR
11	3.74	386.0588	0.8	$C_{12}H_{21}NO_9S_2$	Glucobrassicanapin	367, 306, 274, 259, 190, 95, 74	RP, GP
12	5.84	463.0495	1.9	$C_{16}H_{20}N_2O_{10}S_2$	4-Hydroxyglucobrassicin	426, 393, 323, 294, 239, 206, 169, 116, [illegible]	BR
13	6.54	447.0545	1.7	$C_{16}H_{20}N_2O_9S_2$	Glucobrassicin	377, 320, 252, 225, 205, 161, 120, 96	CK, TK, RP, GP, BR
14	6.62	493.0600	-5.2	$C_{14}H_{26}N_2O_{11}S_3$	Glucorucolamine	357, 342, 247, 229, 173, 96	BR
15	7.12	477.0652	1.9	$C_{17}H_{22}N_2O_{10}S_2$	Neoglucobrassicin	446, 351, 295, 224, 144, 96	CC, CK, TK, RP, GP, BR
				**Isothiocyanates**			
16	0.95	150.0231	0.6	$C_4H_9NO_3S$	3-(Methylsulfinyl)alanine (Methiin)	62	BR

17	1.01	160.0266	3.8	$C_6H_{11}NS_2$	4-(Methylthio)butyl mustard oil (Erucin)	153, 116, 74	CK, TK, RP, GP,
18	2.09	176.0209	0	$C_6H_{11}NOS_2$	Sulforaphane	145	CC, CK, TK, RP, GP, BR
19	3.52	218.0678	-0.3	$C_9H_{17}NOS_2$	1-Isothiocyanato-7-(methylsulfinyl)heptane	102	BR
					**Phenolic acids and conjugates**		
20	2.85	137.0243	-0.7	$C_7H_6O_3$	Salicylic acid	93	CC, TK, RP, GP
21	3.22	147.0449	-1.6	$C_9H_8O_2$	Cinnamic acid	113	CC, CK, TK, RP, GP, BR
22	4.28	315.0722	0.2	$C_{13}H_{16}O_9$	Dihydroxybenzoic acid glucoside	175, 152, 109	CC, CK, TK, RP, GP, BR
23	6.16	503.1399	-1.4	$C_{21}H_{28}O_{14}$	Caffeic acid-di-glucoside	340, 257, 150	BR
24	6.54	179.0354	2.5	$C_9H_8O_4$	Caffeic acid	134	RP, GP
25	6.72	371.0991	2	$C_{16}H_{20}O_{10}$	Hydroxyferulic acid glucoside	163, 119	TK, BR
26	7.03	325.0926	-0.8	$C_{15}H_{18}O_8$	*p*-Coumaric acid glucoside	163, 119	CC, TK, RP, GP
27	7.42	193.0507	0.5	$C_{10}H_{10}O_4$	Ferulic acid	178, 160, 134	CK, TK, BR
28	7.48	353.0880	0.6	$C_{16}H_{18}O_9$	Chlorogenic acid	191, 179, 161, 135	CC, CK, TK, BR
29	7.68	329.0884	1.9	$C_{14}H_{18}O_9$	Vanillic acid glucoside	166	CK, TK, BR
30	7.74	295.0821	-0.4	$C_{13}H_{12}O_8$	Caffeoyl malate	179, 175, 160, 147, 134	RP
31	7.76	355.1037	0.8	$C_{16}H_{20}O_9$	Ferulic acid glucoside	258, 193, 178, 134	CC, CK, TK, GP, BR
32	7.85	385.1143	0.8	$C_{17}H_{22}O_{10}$	Sinapoyl glucoside	223, 179, 164, 149, 119	CC, CK, TK, RP, GP, BR
33	8.00	517.1567	0.9	$C_{22}H_{30}O_{14}$	Ferulic acid dihexoside	355, 193, 175	RP, GP
34	8.03	855.2217	1.9	$C_{37}H_{44}O_{23}$	Diferuloyl triglucoside	693, 646, 605, 560, 427, 335, 296, 247, 192	RP, GP
35	8.12	885.2670	0.0	$C_{39}H_{50}O_{23}$	Sinapoyl feruloyl triglucoside	651, 499, 362, 205, 314, 223, 193, 178	CK
36	8.71	739.2081	-1.3	$C_{33}H_{40}O_{19}$	Sinapoyl hydroxyferuloyl gentiobioside	415, 223, 209, 205, 176, 191, 164, 149, 135	CC, CK, TK, RP, GP, BR
37	8.76	1091.326	0.6	$C_{50}H_{60}O_{27}$	Disinapoyl feruloyl triglucoside	888, 752, 612, 561, 430, 223, 217, 205, 175	CC, RP, GP
38	8.91	341.0879	0.3	$C_{15}H_{18}O_9$	Caffeic acid glucoside	306, 241, 181, 96	TK
39	8.99	223.0611	-0.3	$C_{11}H_{12}O_5$	Sinapic acid	205, 175, 161, 149	CC, CK, TK, RP, GP, BR

40	9.05	309.0613	-0.9	$C_{14}H_{14}O_8$	Feruloyl malate	193, 178, 134	CC, CK, TK, RP, GP, BR
41	9.05	709.1989	0.5	$C_{32}H_{38}O_{18}$	Feruloyl hydroxyl feruloyl gentiobioside	520, 370, 209, 191, 176, 161, 151, 134	CK, TK, RP, GP
42	9.21	753.2236	-1.5	$C_{34}H_{42}O_{19}$	Disinapoyl gentiobiose	223, 205, 175, 190, 161, 149	CK, TK, RP, GP
43	9.23	723.2129	1.7	$C_{33}H_{40}O_{18}$	Feruloyl sinapoyl gentiobioside	699, 650, 607, 561, 505, 461, 399, 223, 193, 191, 175, 164, 134	CK, TK, RP, GP
44	9.87	959.2828	0.2	$C_{45}H_{52}O_{23}$	Trisinapoyl gentiobioside	458, 348, 223, 205, 190, 161, 149	CC, CK, TK, RP, GP, BR
45	10.85	929.2733	1.3	$C_{44}H_{50}O_{22}$	Feruloyl disinapoyl gentiobioside	605, 439, 398, 223, 205, 193, 175, 164, 134	CK, TK, BR
					**Flavonoids**		
46	6.51	463.1248	0.5	$C_{22}H_{24}O_{11}$	Hesperetin glucoside	301, 267, 201, 159	RP, GP
47	6.86	1095.3000	-4.2	$C_{45}H_{60}O_{31}$	Kaempferol-3-triglucoside-7-diglucoside	771, 602, 481, 284, 161	TK
48	6.96	787.1941	0.4	$C_{33}H_{40}O_{22}$	Quercetin-3-*O*-sophoroside-7-*O*-glucoside	462, 299	TK, RP, GP
49	7.04	771.1904	-1.1	$C_{33}H_{40}O_{21}$	Kaempferol-3-*O*-sophoroside-7-*O*-glucoside	609, 446, 284, 183	CC, CK, TK, RP, GP
50	7.14	625.1398	-1.9	$C_{27}H_{30}O_{17}$	Quercetin-3,7-di-*O*-glucoside	462, 299	RP, GP
51	7.20	801.2087	-1	$C_{34}H_{42}O_{22}$	Isorhamnetin 3-sophoroside 7-glucoside (Brassicoside)	639, 493, 476, 449, 434, 394, 315, 251, 178	CK, BR
52	7.20	993.2500	-1.7	$C_{44}H_{50}O_{26}$	Quercetin 3-(2-sinapoylsophoroside) 7-glucoside	655, 625, 462, 299, 209, 178, 127	RP, GP
53	7.21	949.2475	0.8	$C_{39}H_{50}O_{27}$	Quercetin-3-*O*-sophorotrioside -7-*O*-glucoside	787, 462, 299	CC, CK, TK, GP, RP, BR
54	7.22	933.2508	-0.1	$C_{39}H_{50}O_{26}$	Kaempferol-3-*O*-triglucoside-7-*O*-glucoside	771, 591, 372, 284, 269, 257, 178, 161	TK
55	7.24	963.2396	-1.6	$C_{43}H_{48}O_{25}$	Quercetin 3-*O*-(2-feruloylsophoroside) 7-*O*-glucoside	914, 801, 787, 625, 518, 462, 299, 163	TK, GP
56	7.34	933.2307	0.1	$C_{42}H_{46}O_{24}$	Kaempferol 3-(2-caffeoylsophoroside)-7-*O*-glucoside	902, 807, 771, 609, 469, 446, 284, 251, 227, 194, 179, 161	TK, RP, GP

57	7.53	1139.3093	-1	$C_{50}H_{60}O_{30}$	Kaempferol-3-*O*-sinapoyl sophorotrioside-7-*O*-glucoside	977, 771, 753, 591, 447, 284, 223, 205, 191, 164	CK, TK, GP, BR, RP
58	7.62	1109.3010	1.6	$C_{49}H_{58}O_{29}$	Kaempferol-3-*O*-(feruloyl) triglucoside-7-*O*-glucoside	609, 429, 284, 185, 175, 161	TK
59	7.65	977.2580	1.2	$C_{44}H_{50}O_{25}$	Kaempferol 3-*O*-sinapoylglucoside-7-*O*-diglucoside	609, 446, 344, 284, 255, 191	CK, TK, RP, GP
60	7.71	639.1571	0.7	$C_{28}H_{32}O_{17}$	Isorhamnetin 3,7-*O*-diglucoside	476, 313	BR, CK
61	7.75	947.2469	0.7	$C_{43}H_{48}O_{24}$	Kaempferol 3-(feruloyl sophorotrioside)	785, 446, 284, 257, 217, 193, 175, 161, 151	CK, TK, GP, RP, BR
62	7.86	917.2363	0.7	$C_{42}H_{46}O_{23}$	Kaempferol 7-*O*-(6-caffeoyl)-glucosyl rhamnoside-3-*O*-glucoside	721, 476, 363, 284, 178, 163, 135	RP, GP
63	8.23	593.1509	-0.5	$C_{27}H_{30}O_{15}$	Kaempferol 3-*O*-rhamnoside-7-*O*-glucoside	446, 431, 285, 241, 124	CC, CK, TK, GP, BR
64	8.24	609.1438	-3.7	$C_{27}H_{30}O_{16}$	Kaempferol-3-*O*-digucoside	447, 285, 283	CK, TK, GP, RP, BR
65	8.62	463.0866	-3.4	$C_{21}H_{20}O_{12}$	Quercetin-7-*O*-glucoside	301	CC, CK, TK, GP, RP, BR
66	8.82	563.1407	0.2	$C_{26}H_{28}O_{14}$	Kaempferol-*O*-rhamnoside-*O*-[illegible]	431, 285	BR
67	9.07	679.1523	1.1	$C_{30}H_{32}O_{18}$	Kaempferol 3-(6-*O*-malonyl neohesperidoside)	285	CC, RP
					**Organic and Fatty acids**		
68	0.86	115.0036	-0.5	$C_4H_4O_4$	Fumaric acid	92, 71	CC, CK, TK, RP, GP, BR
69	1.08	133.0142	-0.2	$C_4H_6O_5$	Malic acid	115, 71	CC, CK, TK, RP, GP, BR
70	1.44	191.0196	-0.5	$C_6H_8O_7$	Citric acid	111, 87, 76, 57	CC, CK, TK, RP, GP, BR
71	1.66	117.0194	0.8	$C_4H_6O_4$	Succinic acid	99	CC, CK, TK, RP, GP, BR
72	6.57	205.0348	-2.7	$C_7H_{10}O_7$	Methyl citric acid	161, 87	BR
73	7.38	191.0561	0.1	$C_7H_{12}O_7$	Quinic acid	165, 155, 147, 127, 109	CK, RP
74	7.84	215.1291	1.1	$C_{11}H_{20}O_4$	Undecanedioic acid	153, 136	CC, CK, GP
75	8.03	173.0454	-0.7	$C_7H_{10}O_5$	Shikimic acid	158, 93, 85	TK
76	8.72	163.0400	-0.3	$C_9H_8O_3$	Hydroxy nonenediynoic	119	GP
77	8.98	193.0504	-1.1	$C_{10}H_{10}O_4$	Decatetraenedioic acid	148, 135, 111, 89	CC

78	9.49	187.0974	-0.8	$C_9H_{16}O_4$	Nonanedioic acid	169, 125, 97	CC, CK, TK, RP, GP, BR
79	10.23	255.2327	-0.9	$C_{16}H_{32}O_2$	Palmitic acid	233, 198, 167	CC, CK, TK, RP, GP, BR
80	10.43	201.1133	0.4	$C_{10}H_{18}O_4$	Dihydroxy decenoic acid	139	TK, RP, GP
81	10.97	211.1338	-0.7	$C_{12}H_{20}O_3$	Hydroxy dodecadienoic acid	184	CC, CK, TK, RP, GP, BR
82	11.05	225.1133	0.4	$C_{12}H_{18}O_4$	Hydroxyjasmonic acid	184, 113	RP, GP
83	11.46	329.2331	-0.7	$C_{18}H_{34}O_5$	Trihydroxy octadecenoic acid	293, 201, 171	CC, CK, TK, RP, GP, BR
84	12.04	279.2325	-1.5	$C_{18}H_{32}O_2$	Linoleic acid	201, 175, 136, 117	CC, CK, TK, RP, GP, BR
85	12.72	343.2296	5.1	$C_{22}H_{32}O_3$	Hydroxy docosahexaenoic acid	312, 263, 214, 171, 151	CK, RP
86	14.59	315.2533	-2.4	$C_{18}H_{36}O_4$	Dihydroxy stearic acid	297, 276, 200, 171, 119	BR
87	14.73	313.2014	-2	$C_{17}H_{30}O_5$	Trihydroxy heptadecadienoic acid	257, 208, 171, 142, 99	CK, TK
88	15.09	275.2020	1.3	$C_{18}H_{28}O_2$	Stearidonic acid	162, 129, 114	CC, CK, TK, RP, GP, BR
89	15.83	295.2276	-0.8	$C_{18}H_{32}O_3$	Hydroxy octadecadienoic acid	295, 277, 183	CK, TK, RP, BR
90	16.58	297.2433	-0.7	$C_{18}H_{34}O_3$	Hydroxy oleic acid	279, 239, 183, 155	CC, RP, GP, BR
91	16.80	205.1596	-0.8	$C_{14}H_{22}O$	Tetradecatrienal	197, 126, 69	CC, TK
92	17.02	249.1857	-1.1	$C_{16}H_{26}O_2$	Hexadecatrienoic acid	205, 154, 112	CK, TK, RP, GP
93	17.06	311.2224	-1.2	$C_{18}H_{32}O_4$	Dihydroxy octadecadienoic acid	223, 208, 151, 128, 87, 57	CC, TK, GP, BR
94	17.09	149.0971	-0.4	$C_{10}H_{14}O$	Decatrienal	149	CC, TK
95	18.07	251.2017	0.3	$C_{16}H_{28}O_2$	Hexadecadienoic acid	207, 183, 122	TK
96	18.44	277.2172	-0.3	$C_{18}H_{30}O_2$	Octadecadienoic acid	210, 159, 54	CK, TK, RP, GP, BR
97	18.46	381.3377	0.8	$C_{24}H_{46}O_3$	Hydroxy tetracosenoic acid	335, 162, 118	CK
98	18.50	271.2277	-0.5	$C_{18}H_{32}O_3$	Hydroxy palmitic acid	225, 170, 140	CK, TK, GP, BR
99	20.11	327.2909	-0.7	$C_{20}H_{40}O_3$	Hydroxy eicosanoic acid	183	GP, BR

*Retention times and m/z errors are an average value for the different species. **MS/MS fragments were a combination of all the fragments detected in the different speces. [a] CC, Chinese cabbage, CK, Curly kale, TK, Tuscan kale, RP, red Pak choi, GP, green Pak choi, BR, Brussel sprouts.

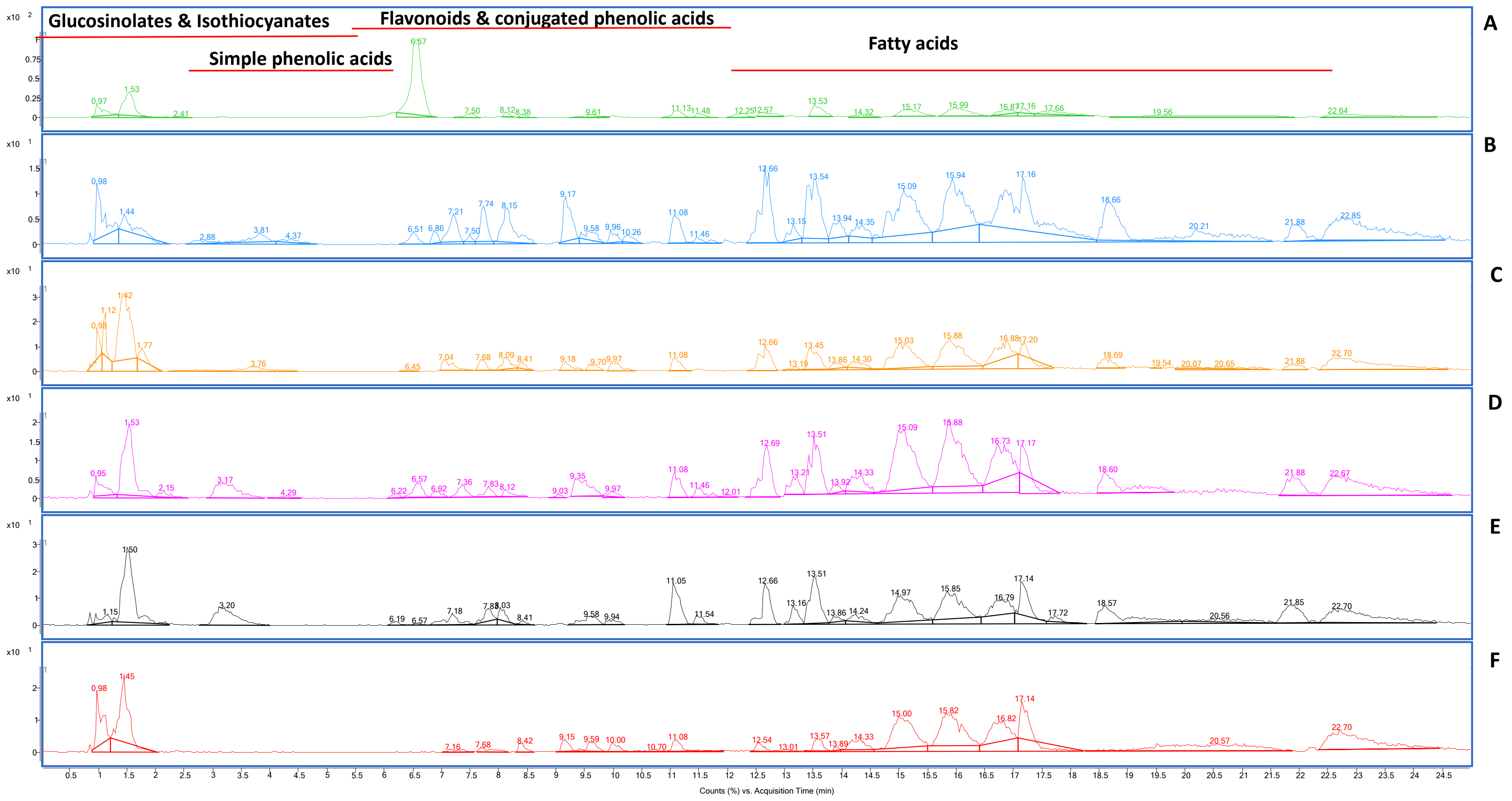

Figure S1. LC-QTOF-MS base peak chromatograms of the methanolic leaf extracts of the six *Brassica* species analyzed in the negative ESI mode. A, Chinese cabbage (CC); B, Curly kale (CK); C, Tuscan kale (TK); D; red Pak choi (RP); E, green Pak choi (GP); F, Brussel sprouts (BR)

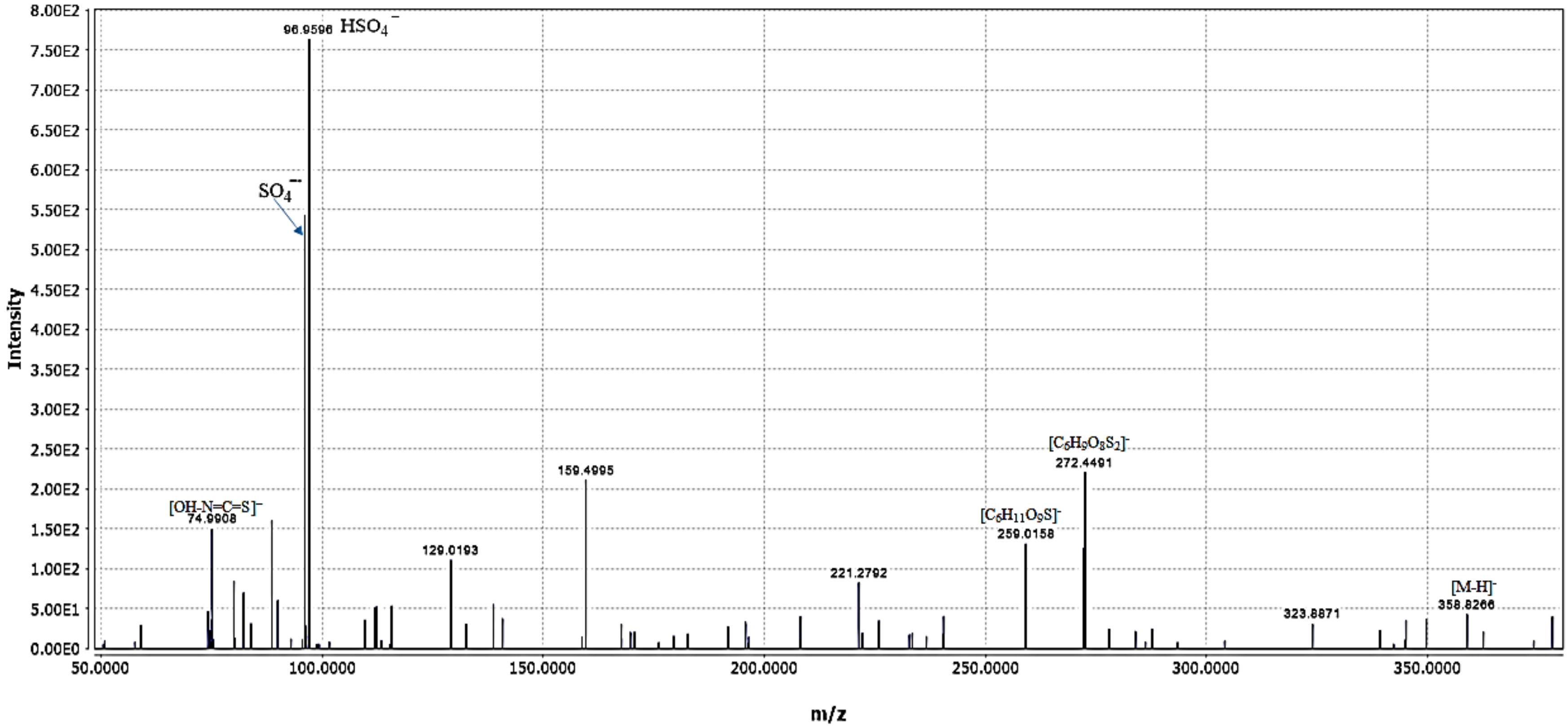

Figure S2. MS/MS spectrum of sinigrin (ID number **1**, Table S1)

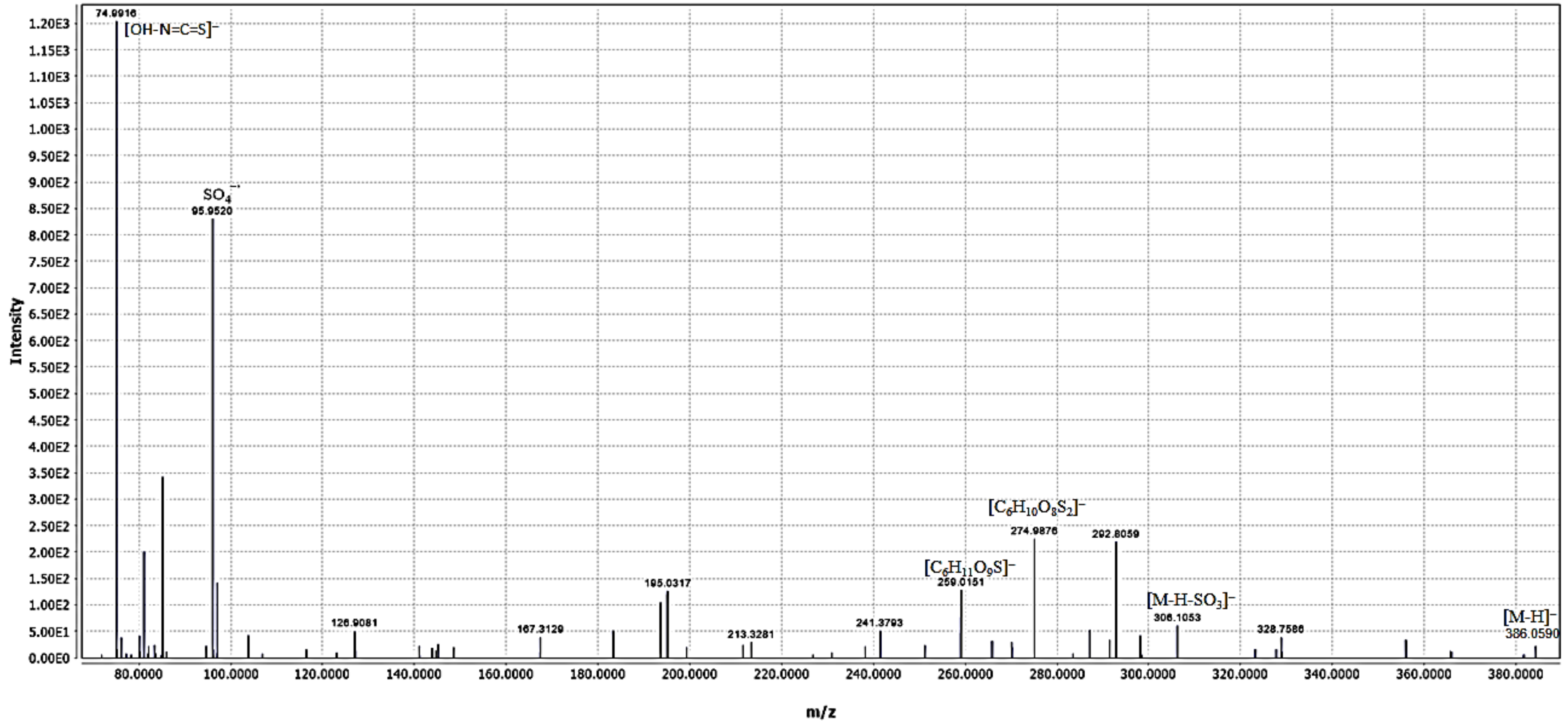

Figure S3. MS/MS spectrum of glucobrassicanapin (ID number **11**, Table S1)

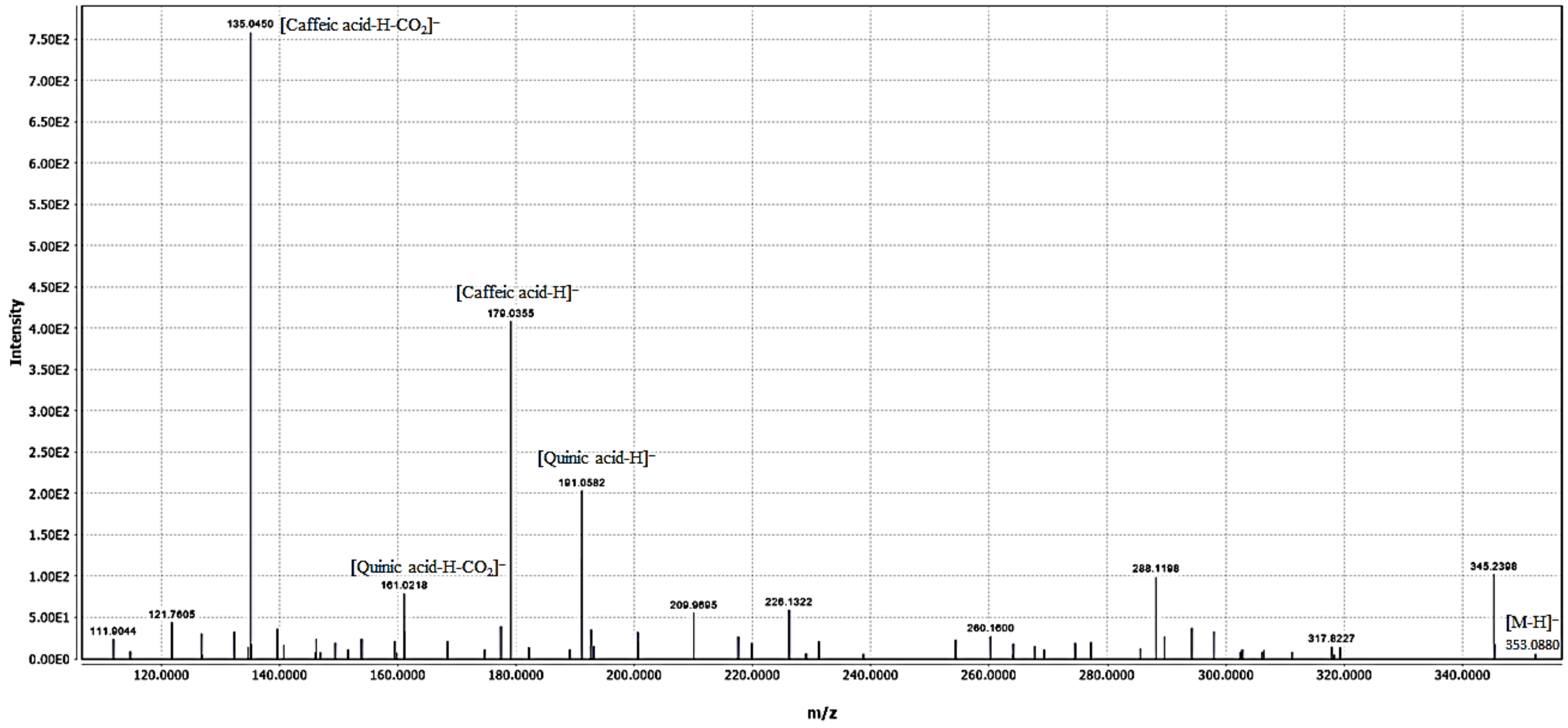

Figure S4. MS/MS spectrum of chlorogenic acid (ID number **28**, Table S1)

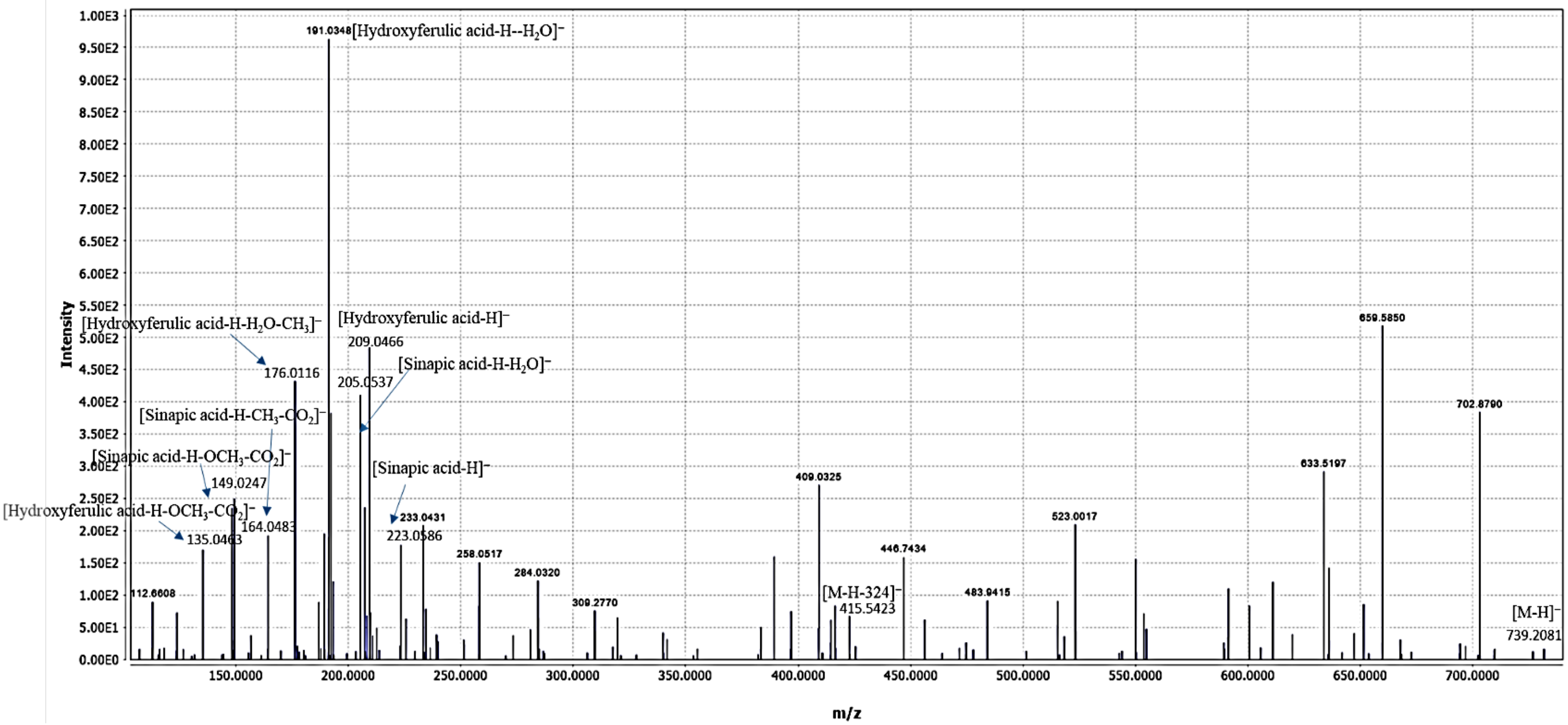

Figure S5. MS/MS spectrum of sinapoyl hydroxy feruloyl gentiobioside (ID number **36**, Table S1)

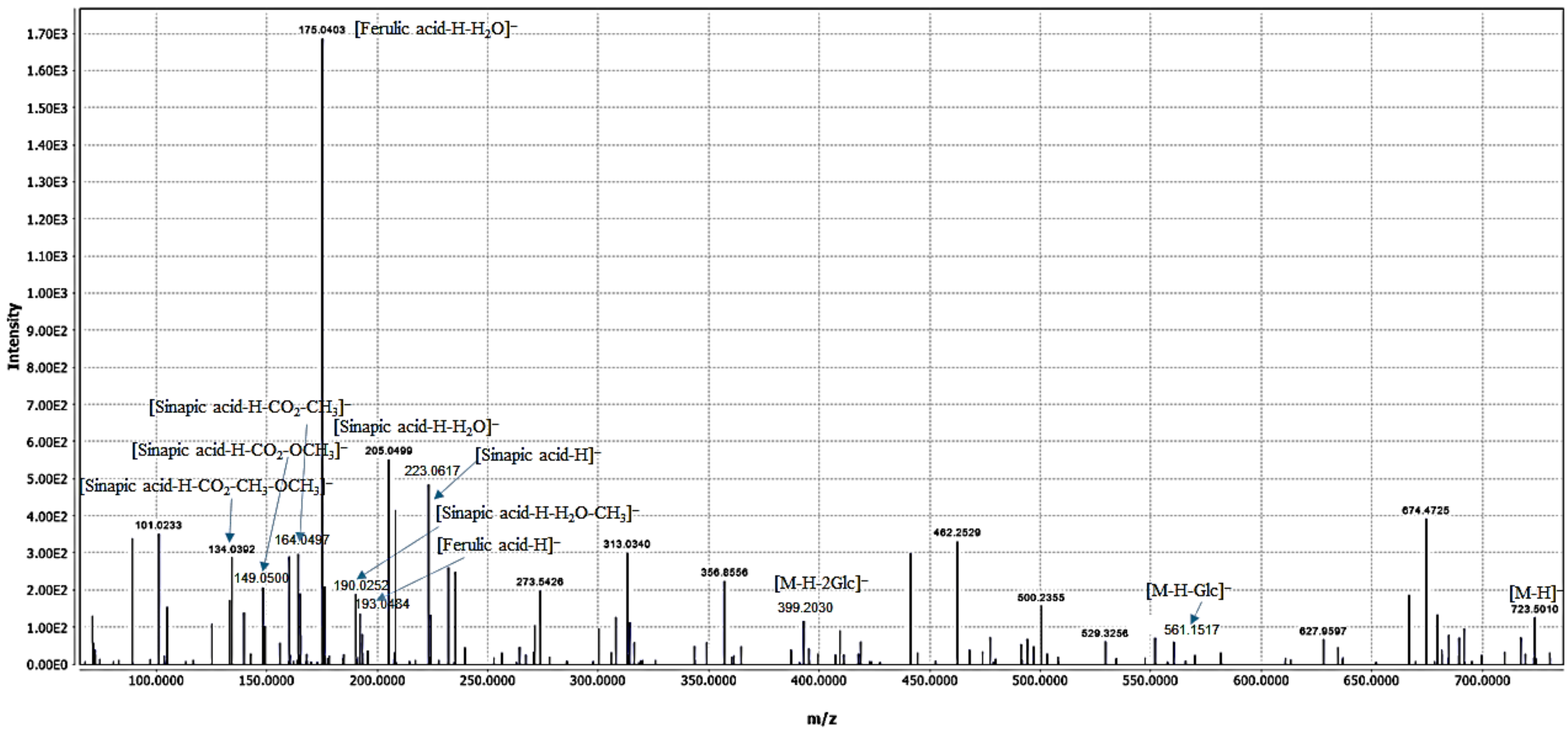

Figure S6. MS/MS spectrum of feruloyl sinapoyl gentiobioside (ID number **43**, Table S1)

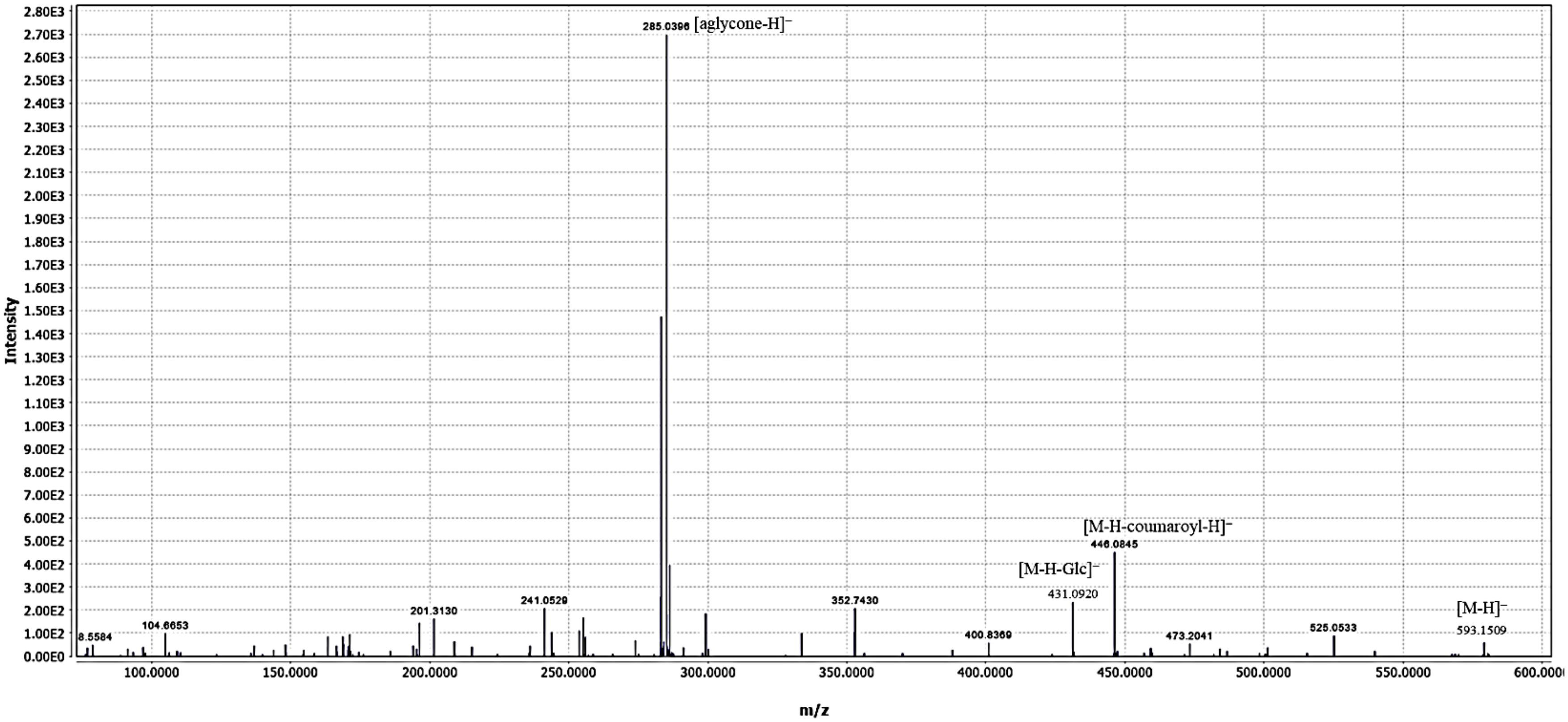

Figure S7. MS/MS spectrum of kaempferol-3-*O*-rhamnosyl-7-*O*-hexoside (ID number **63**, Table S1)

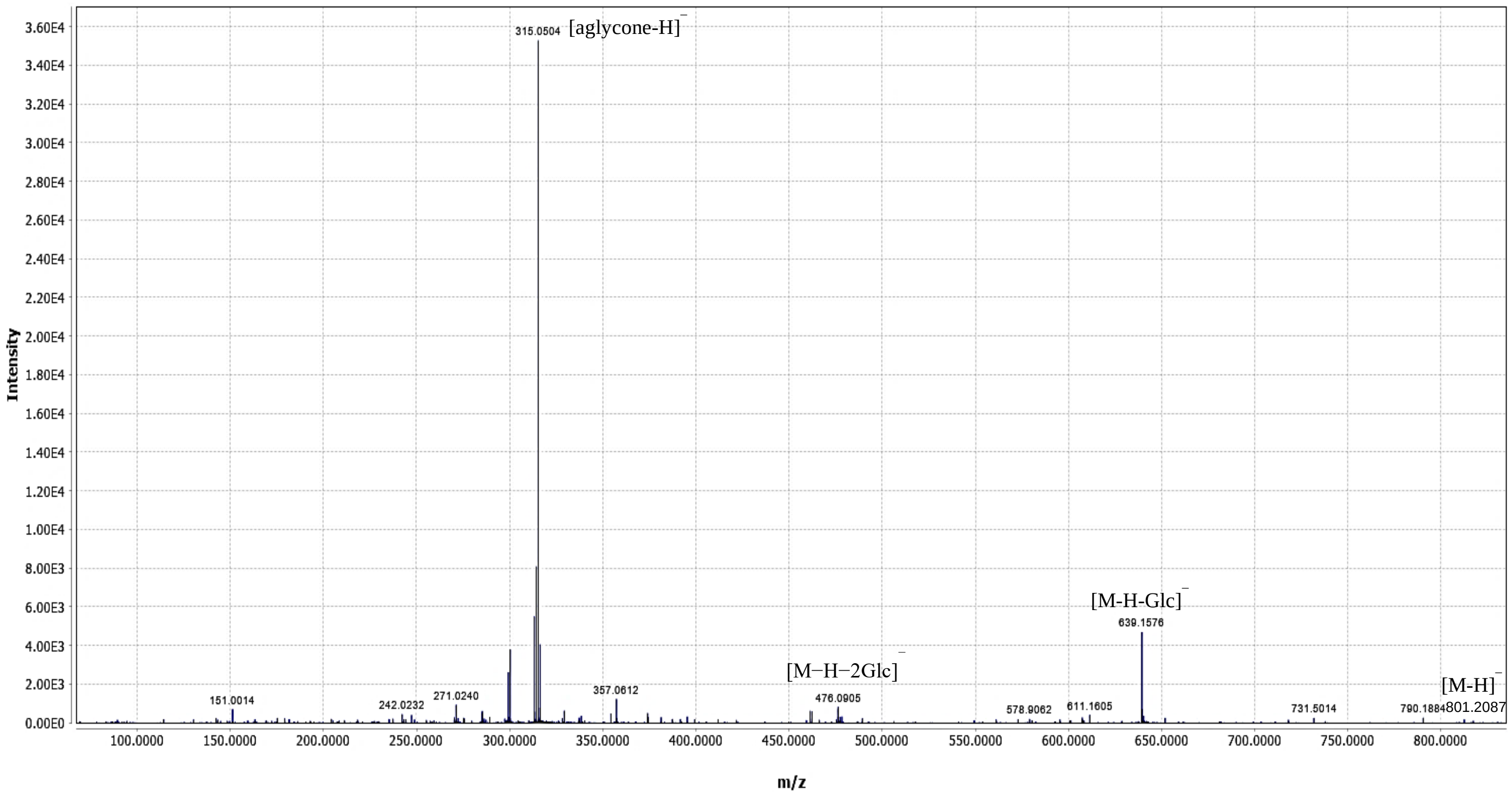

Figure S8. MS/MS spectrum of isorhamnetin 3-sophoroside 7-glucoside (Brassicoside) (ID number **51**, Table S1)

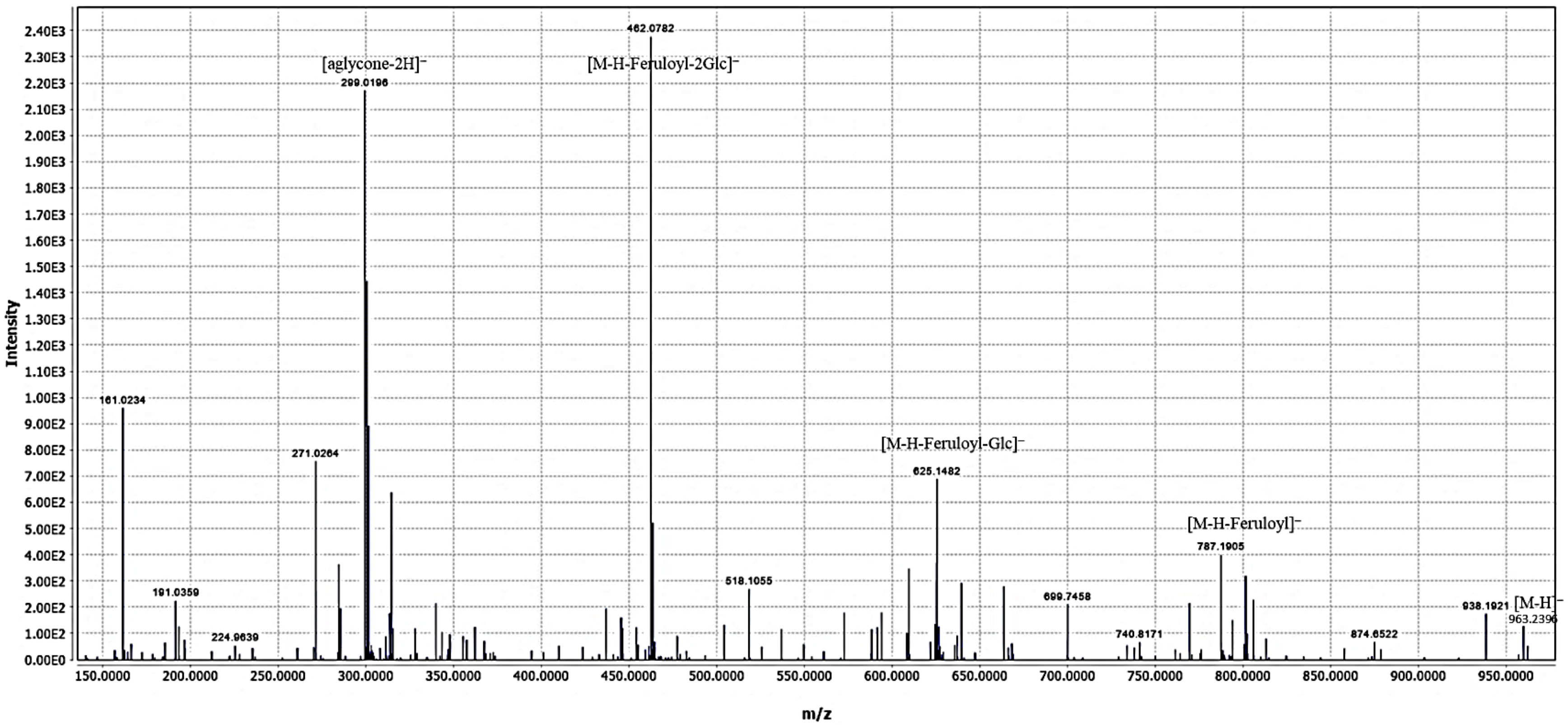

Figure S9. MS/MS spectrum of quercetin 3-*O*-(2-feruloylsophoroside)-7-*O*-glucoside (ID number **55**, Table S1)

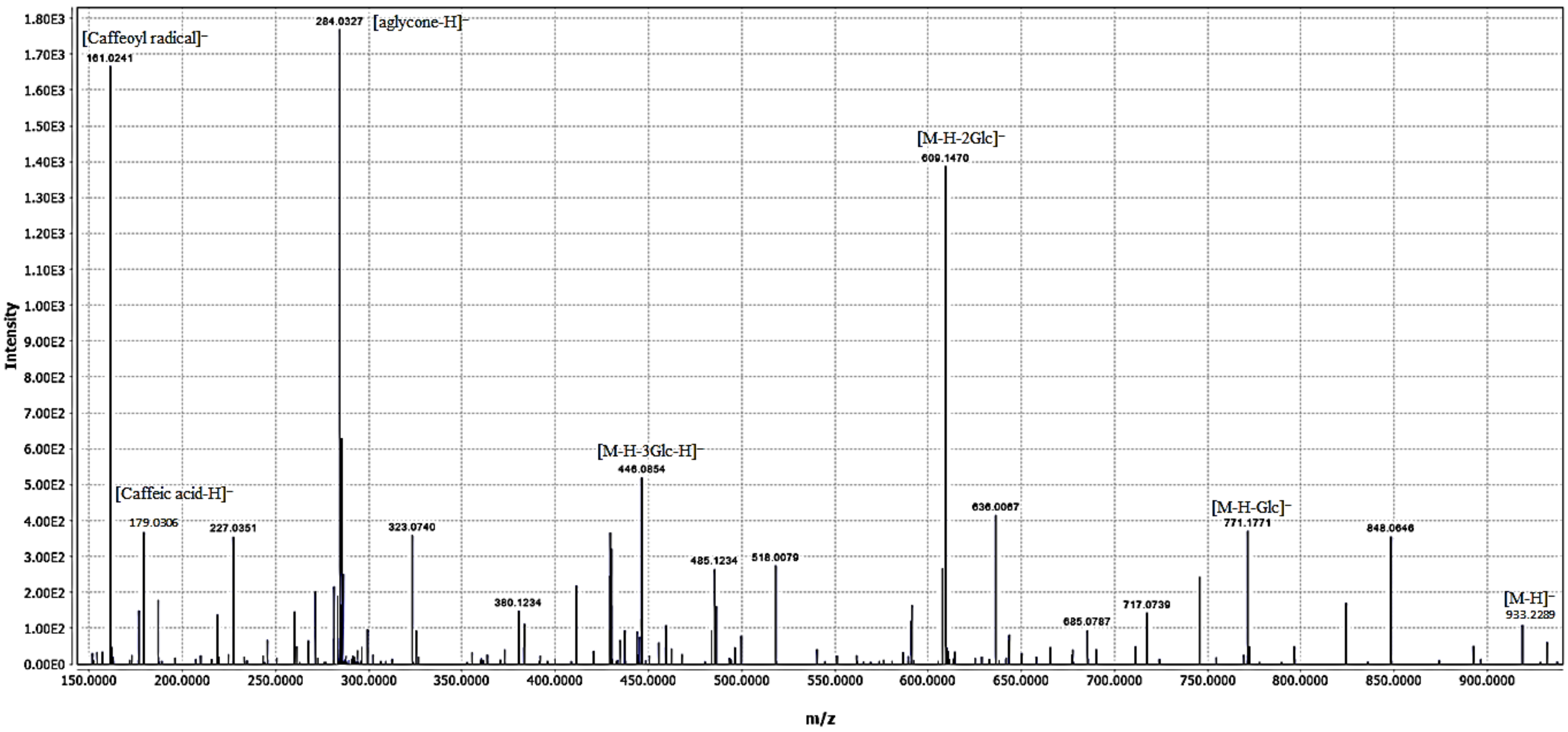

Figure S10. MS/MS spectrum of kaempferol 3-(2-caffeoylsophoroside)-7-*O*-glucoside (ID number **56**, Table S1)

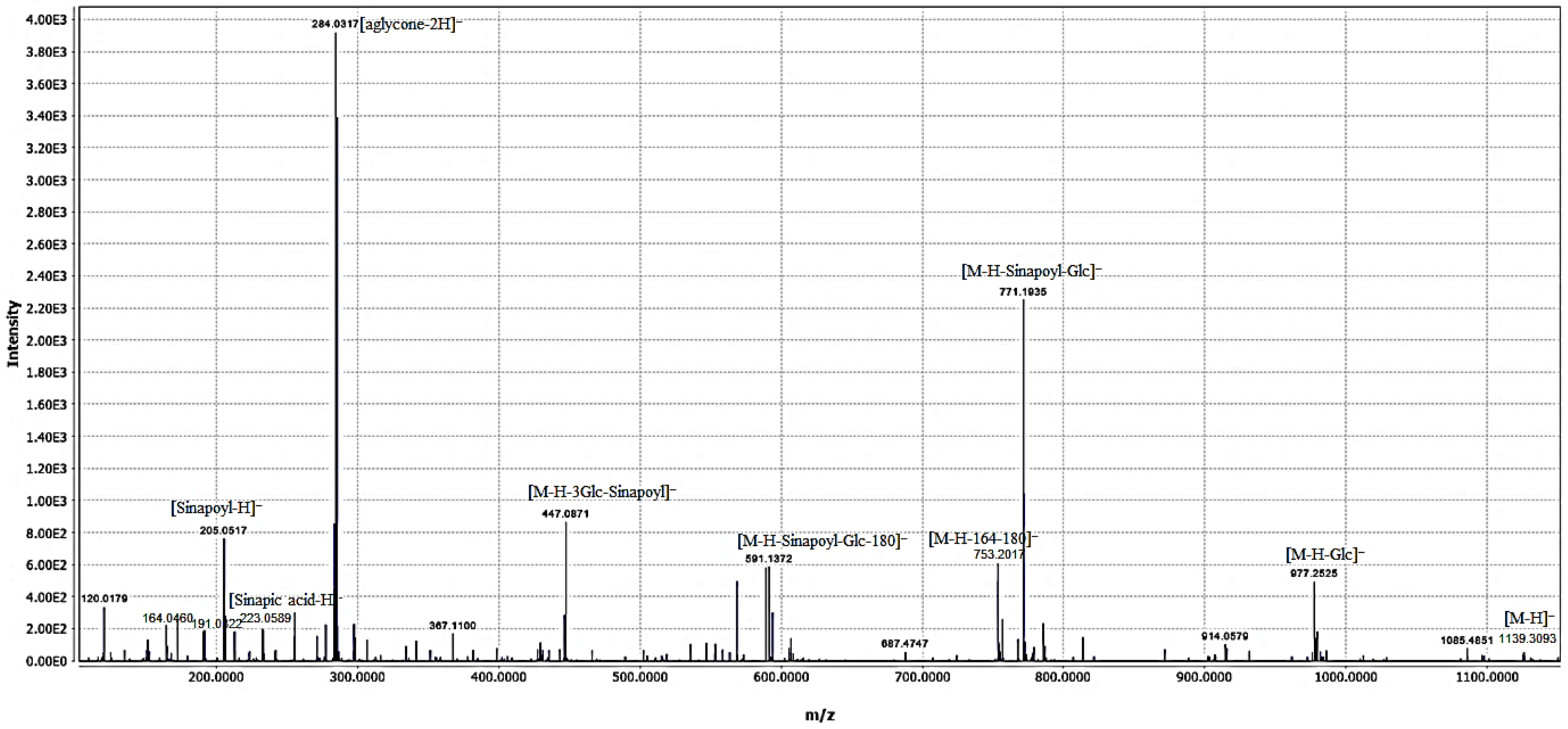

Figure S11. MS/MS spectrum of kaempferol-3-*O*-sinapoyl sophorotrioside-7-*O*-glucoside (ID number **57**, Table S1)

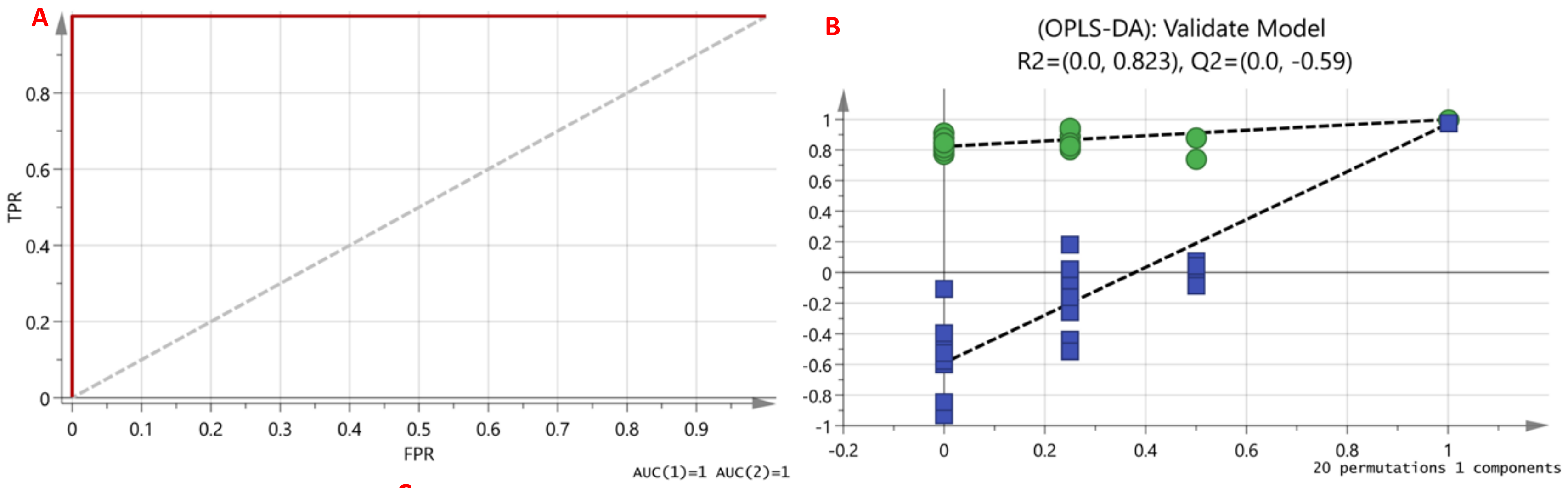

C

M1	SS	DF	MS	F	p	SD
Total corr.	17	17	1			1
Regression	16.4949	6	2.74915	59.8692	9.24687e-008	1.65806
Residual	0.505111	11	0.0459192			0.214288

Fig. S12. OPLS-DA optimization and validation parameters for modeling active extracts (●) against inactive ones (●) based on the whole LC-MS data set and the bioactivity results, *n*=3. (A) The receiver operating characteristic (ROC) curve to assess the classification ability of the developed OPLS-DA showing AUC = 1. (B) Permutation test, *n* = 20 that showed negative Q2 intercept value. (C) CV-ANOVA to assess for model statistical significance.

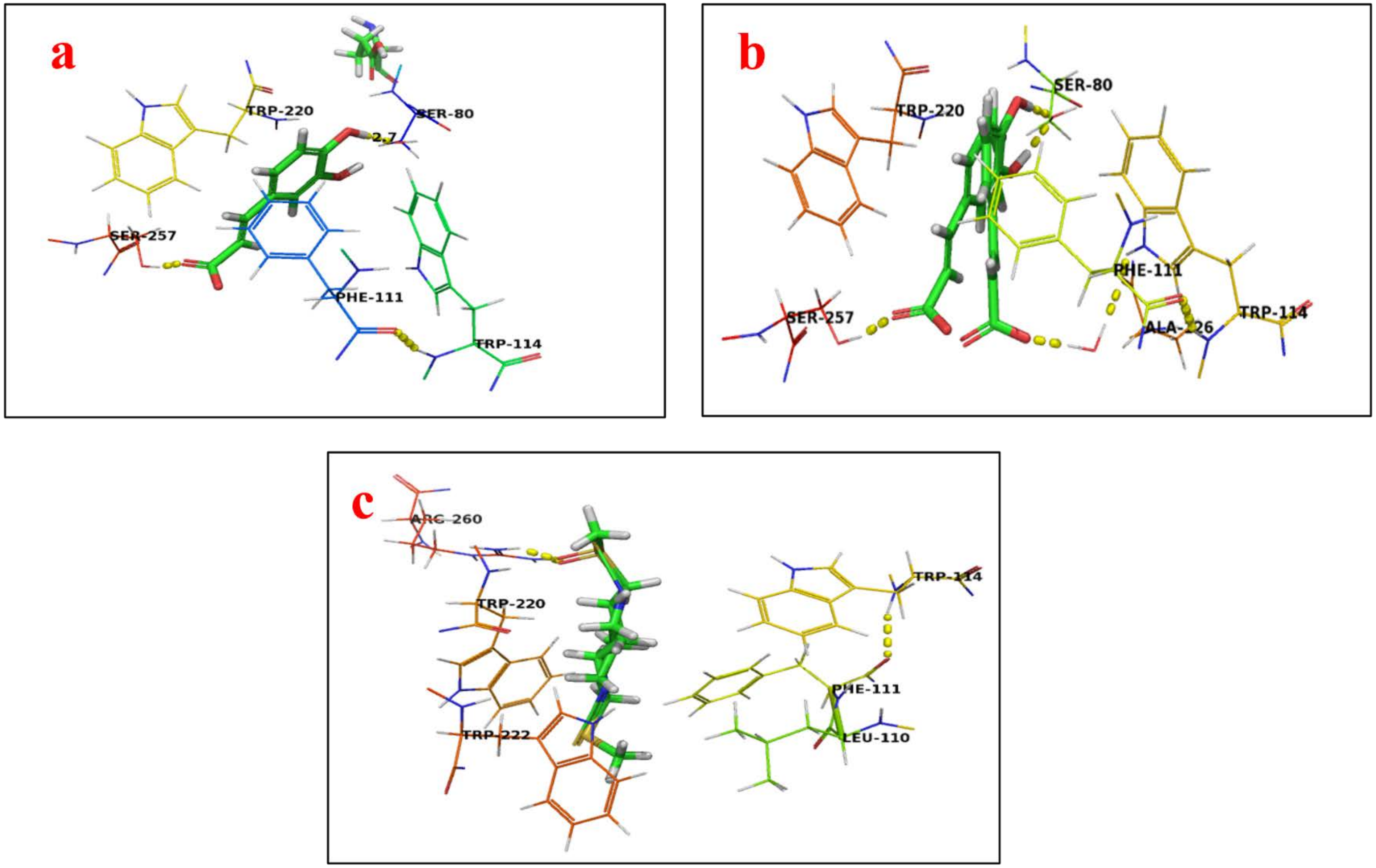

Figure S13. Crystal structure of *A. baumannii* lipase receptor with **a:** caffeic acid (ID 24).; **b**: aligned cinnamic acid (ID 21) and caffeic acid (ID 24); **c:** aligned erucin (ID 17) and sulforophane (ID 18); showing similar binding modes betwwen the two phenolic acids and the two isothiocyanates. The hydrogen bonding interactions are presented by dashed lines. Residues contacting ligand are demonstrated as lines.
